# Modeling the early stages of Alzheimer’s disease by administering intracerebroventricular injections of human native Aβ oligomers to rats

**DOI:** 10.1186/s40478-022-01417-5

**Published:** 2022-08-16

**Authors:** Eva Baerends, Katia Soud, Jonas Folke, Anna-Kathrine Pedersen, Simon Henmar, Lisa Konrad, Matthew D. Lycas, Yuki Mori, Bente Pakkenberg, David P. D. Woldbye, Oksana Dmytriyeva, Stanislava Pankratova

**Affiliations:** 1 Department of Neuroscience, Faculty of Health and Medical Sciences, University of Copenhagen, 2200 Copenhagen, Denmark; 2 Centre for Neuroscience and Stereology, Department of Neurology,, Bispebjerg-Frederiksberg Hospital, Copenhagen University Hospital, Copenhagen, Denmark; 3 Copenhagen Center for Translational Research, Bispebjerg-Frederiksberg Hospital, Copenhagen University Hospital, Copenhagen, Denmark; 4 Proteomics Program, Novo Nordisk Foundation Center for Protein Research, Faculty of Health and Medical Sciences, University of Copenhagen, Copenhagen, Denmark; 5 Center for Translational Neuromedicine, Faculty of Health and Medical Sciences, University of Copenhagen, Copenhagen, Denmark; 6 Department of Clinical Medicine, Faculty of Health and Medical Sciences, University of Copenhagen, Copenhagen, Denmark; 7 Novo Nordisk Foundation Center for Basic Metabolic Research, Faculty of Health and Medical Sciences, University of Copenhagen, Copenhagen, Denmark; 8 Comparative Pediatrics and Nutrition, Department of Veterinary and Animal Sciences, University of Copenhagen, Copenhagen, Denmark

**Keywords:** Alzheimer’s disease, Social recognition, Hippocampus, Lateral entorhinal cortex, Neuroinflammation

## Abstract

Alzheimer’s disease (AD) is a progressive and irreversible neurodegenerative disease characterized by the accumulation of aggregated amyloid beta (Aβ) and hyperphosphorylated tau along with a slow decline in cognitive functions. Unlike advanced AD, the initial steps of AD pathophysiology have been poorly investigated, partially due to limited availability of animal models focused on the early, plaque-free stages of the disease. The aim of this study was to evaluate the early behavioral, anatomical and molecular alterations in wild-type rats following intracerebroventricular injections of human Aβ oligomers (AβOs). Bioactive human AD and nondemented control brain tissue extracts were characterized using ELISA and proteomics approaches. Following a bilateral infusion, rats underwent behavioral testing, including the elevated plus maze, social recognition test, Morris water maze and Y-maze within 6 weeks postinjection. An analysis of brain structure was performed with manganese-enhanced MRI. Collected brain tissues were analyzed using stereology, immunohistochemistry, ELISA and qPCR. No sensorimotor deficits affecting motor performance on different maze tasks were observed, nor was spatial memory disturbed in AD rats. In contrast, a significant impairment of social memory became evident at 21 days postinjection. This deficit was associated with a significantly decreased volume of the lateral entorhinal cortex and a tendency toward a decrease in the total brain volume. Significant increase of cleaved caspase-3-positive cells, microglial activation and proinflammatory responses accompanied by altered expression of synaptic markers were observed in the hippocampus of AD rats with immunohistochemical and qPCR approaches at 6 weeks postinjection. Our data suggest that the social memory impairment observed in AβO-injected rats might be determined by neuroinflammatory responses and synaptopathy. An infusion of native oligomeric Aβ in the rat brain represents a feasible tool to model early plaque-free events associated with AD.

**Supplementary Information:**

The online version contains supplementary material available at 10.1186/s40478-022-01417-5.

## Introduction

Alzheimer’s disease (AD) is the most common form of dementia and is characterized by a progressive decline in cognitive and sociobehavioral functions over time. Cerebral accumulation of amyloid beta (Aβ) peptides is one of the key hallmarks of this form of dementia. In contrast to early onset familial AD (FAD), which accounts for less than 5% of all AD cases, late-onset sporadic AD (sAD) accounts for most AD cases and differs from FAD in several neuropathological and clinical features (reviewed in [1]). sAD is initiated and may develop asymptomatically for decades before cognitive impairment appears [2]; thus, proper models reflecting the early, plaque-free stages of sAD are very important.

Transmembrane amyloid precursor protein (APP) is abundantly expressed in the central nervous system and is critical for brain developmental and physiological processes [3–5]. Under pathological conditions, short Aβ peptides of various lengths (36–43 amino acids) are liberated, where 40–42 amino acid peptides are preponderant [6]. These APP cleavage products tend to form aggregates of different complexity, ranging from soluble multimeric nonfibrillary aggregates, called Aβ oligomers (AβOs), to tightly packed amyloid fibrils—the main component of amyloid plaques. Importantly, AβOs appear early in the disease and are considered the primary toxic form of Aβ [7, 8]. The levels of soluble AβOs correlate strongly with synaptic loss, particularly in the entorhinal cortex (EC) [9], and with the severity of cognitive deficits [10]. Mechanistically, the neurotoxic and synaptic deficits induced by AβOs are mediated by the inhibition of long-term potentiation (LTP), decreased synaptic glutamate uptake and spine density, increased intracellular Ca^2+^ concentrations, the induction of inflammation and oxidative stress, disruption of lipid bilayers and promotion of amyloid seeding when injected in different transgenic (Tg) AD mice [8, 11, 12]. Thus, according to the Aβ hypothesis, AβOs likely create a favorite condition for AD onset and development [2, 7, 13, 14].

Although the soluble AβOs appear to play a crucial role in disease development [2], models closely replicating the initial plaque-free stages of AD are limited. Currently, Tg mice and, to a lesser extent, Tg rats represent the majority of animal models used in AD research (reviewed in [1, 15, 16]). However, genetically induced Aβ overproduction mainly simulates FAD pathology and does not fully recapitulate the pathological hallmarks of human AD unless an additional transgene is introduced [17, 18]. In addition, Tg animals produce artificially higher levels of APP, and the presence of its cleavage products early in life may interfere with normal brain development or learning and memory [16]. Consequently, different animal models designed to mimic sAD have been developed in wild-type (WT) animals through viral delivery of Aβ_40/42_ or central treatment with synthetic Aβ peptides [19–21]. Although the synthetic Aβ peptides applied in vivo, such as Aβ_25–35_, Aβ_1–40_ and Aβ_1–42_, are a useful tool, they do not closely replicate the conformational heterogeneity and neurotoxic profile of native AβO species isolated from the brains of patients with sAD (reviewed in [8]) or Tg mice [12]. More than a decade ago, a single intraventricular injection of native soluble AβOs in healthy rats was shown to impair memory and decrease synaptic density, thus providing the concept of a novel inducible sAD model that addresses this methodological gap [7, 14]. Since then, native AβOs have been prepared as Tris-buffered saline (TBS), phosphate-buffered saline (PBS) [22] or artificial cerebrospinal fluid (CSF) [23, 24] extracts from brain tissue homogenates of either postmortem human samples or Tg mice overexpressing human APP. This concept has been increasingly employed to mimic AD pathology in physiologically normal, non-Tg rodents (reviewed in [8]) to investigate the mechanisms of AβO neurotoxicity and the effect on memory. However, in these models, memory and cognitive functions were investigated in a short time interval after the infusion, usually within 24 h to 7 days, and were assessed using few memory tests [7, 14]. Moreover, synaptic dysfunction induced by an infusion of oligomeric Aβ has been described as a relatively acute neurotoxic effect occurring within minutes or hours [25]. Researchers have not addressed whether exogenous AβOs may contribute to persistent AD-related pathology.

Here, we investigated the effect of a bilateral intracerebroventricular (i.c.v.) infusion of native AβOs obtained from human sAD brain tissues on cognitive and memory functions of adult male rats 2.5–6 weeks after the injection followed by MRI and ex vivo tissue analyses.

## Methods

### Human brain samples

Snap-frozen, postmortem neocortical samples (prefrontal cortex: gyrus medialis frontalis) from six patients with severe AD dementia (age 77.7 ± 9.8 years, five females (F)/one male (M), Braak stages 4–6) and five age-matched nondemented controls (age 81.8 ± 6.5 years, two F/three M, Braak stages 1–3) were generously donated by The Netherlands Brain Bank (National Institute for Neuroscience, Amsterdam, The Netherlands). The demographic and clinical characteristics of each individual subject and the statistical comparison between the AD and nondemented control groups are listed in Additional file 5: Table S1. All AD cases were neuropathologically confirmed [26]. Only tissues from subjects older than 60 years with sporadic AD according to their journal’s records were chosen.

### Preparation of the soluble AβO extract and ELISA

The extraction of buffer-soluble AβOs from human brain samples was performed as described previously [25] with minor modifications. Frozen brain samples were weighed, thawed on ice, mixed with five volumes of freshly prepared homogenization buffer (Ca^2+^- and Mg^2+^-free PBS, pH 7.4, protease inhibitor cocktail (Roche Diagnostics, Mannheim, Germany)) and homogenized with 10–15 strokes of a Teflon pestle in a glass homogenizer. The homogenate was incubated on ice for 25–30 min and then centrifuged at 9000×*g* for 15 min at 4 °C to pellet tissue debris. The supernatant was transferred to a new tube and centrifuged at 20,000×*g* for 20 min at 4 °C, and the protein concentration was measured using the Pierce BCA Protein Assay kit (Thermo Fisher Scientific). Equal amounts of cortical tissue extracts from the AD and nondemented controls were pooled, resulting in two pooled groups designated AD and non-AD control extracts, respectively, with a 3.32 mg/ml protein concentration in each sample. These samples were aliquoted and stored at − 80 °C until use. The levels of water-soluble Aβ species and phosphorylated tau (pTau) were quantified in both individual and pooled samples with the triplex Aβ Peptide Panel 1 (4G8) V-PLEX Kit (Meso Scale Diagnostics, Rockville, MD, USA) and pτ ELISA kit (Elabscience), respectively, according to the manufacturer’s protocols. The ELISA plate used to measure the levels of Aβ species was read using an MSD Sector Imager S600 (Meso Scale Diagnostics), while the ELISA plate used to measure pTau was analyzed using a Multiskan FC Microplate Photometer (Thermo Fisher Scientific) at 450/620 nm.

### Liquid Chromatography tandem mass spectrometry (LC–MS/MS) measurements and data analysis

Two technical replicates of pooled AD and non-AD control extract samples were lysed in boiling 6 M guanidine-HCl, 100 mM Tris (pH 8.5), 5 mM TCEP, and 10 mM CAA and processed essentially as described elsewhere [27] with automated PAC digestion using a KingFisher™ Flex robot (Thermo Fisher Scientific). Peptides were eluted from Sep-Pak C18 cartridges with 40% acetonitrile and 0.1% formic acid and analyzed with an on-line nanoflow LC–MS/MS system using Easy nLC1200 (Thermo Fisher Scientific) coupled to a Q-Exactive HF-X mass spectrometer (Thermo Fisher Scientific) through a nanoelectrospray source as described previously [28]. Raw MS data were analyzed using MaxQuant software version 1.6.5.0 with the integrated Andromeda search engine [29] and searched against a target/decoy version of the human UniProt database (release 2019_01). Proteomics data analysis was performed using Perseus software version 1.6.2.2 [30]. Identified proteins were filtered for contaminants and reversed hits, and only proteins identified in all four samples were included in the subsequent analysis. Log2-transformed intensities were normalized by quantile-based normalization. Significantly regulated proteins were determined using Student’s *t*-test (significance cutoff 0.01 and s0 = 0.1), and the results of the analysis were visualized by constructing a volcano plot.

### Animals

Adult male Wistar rats (380–400 g; ~ 3-month-old at arrival; Janvier) were housed in groups of four per large polycarbonate cage (80 × 60 × 60 cm), and housed under standard climate-controlled housing conditions with a 12 h light cycle and free access to water and rat chow. After one week of acclimation, rats were habituated and randomly allocated to two groups. All parts of the in vivo study, including the stereotactic injections, behavioral tests, MRI scanning and the following histological analyses, were performed by investigators who were blinded to the treatment. Injections and all behavioral experiments were performed with 12 rats per group. At the end of the in vivo study, 6 rats from each group were randomly selected for MRI analysis, whereas another 6 rats from each group were used for ex vivo analysis.

### Stereotactic injections

Rats were deeply anesthetized with 4% isoflurane (Attane, ScanVet, Denmark) and placed in a stereotaxic frame (Kopf Instruments, Tujunga, CA, USA), where the anesthesia was maintained with 1.5–2% isoflurane mixed with a 0.8 L/min oxygen flow throughout surgery. Lidocaine (Xylocain, AstraZeneca, Denmark) was applied locally on the head before cutting the skin. Eight microliters of AD or non-AD control brain tissue extracts were infused i.c.v. into each hemisphere at the following coordinates from the bregma: anterior/posterior—1 mm, medial/lateral ± 1.5 mm, and dorsal/ventral 4 mm. The injections were performed with a 10 µl Hamilton syringe (Hamilton Company, USA) fitted with a thin glass capillary cannula. The infusion speed was approximately 0.5 µl/min, and the needle was left in place for 3 min followed by slow retraction. For analgesia, buprenorphine (0.05 mg/kg) and carprofen (5 mg/kg) were administered subcutaneously prior to and for two days after the surgery.

### Behavioral tests

After 2.5 weeks of postsurgical recovery, rats were subjected to a battery of behavioral tests within the next 3.5 weeks, followed by MRI scanning and tissue collection (see Fig. 2 for the study design). All behavioral experiments were conducted during the light cycle, and rats were habituated to the experimenters and allowed to habituate to the test room for 1–2 h prior to the start of each behavioral test. Body weight was measured weekly and showed no difference between groups during the study (data not shown). Throughout the study, rats did not show any signs of aggressive behavior.

### Elevated plus maze (EPM)

The EPM test was conducted on Day 17 after the injections to assess the anxiety state, as previously described [31]. Each arm was 45/15/70 cm in length/width/distance from the ground, while the closed arms were enclosed by 15 cm high opaque walls. Each rat (n = 12 per group) was placed at the center of the platform facing an open arm and allowed to freely explore the platform for 10 min. Sessions were filmed and analyzed with DeepLabCut software [32, 33]. The time spent in the open and closed arms and the central square, number of entries, speed and latency to first entrance in open arms were analyzed. The entry was considered valid when all four paws and the tail base entered the arm.

### Social recognition test (SRT)

Social recognition memory was evaluated on Day 21 after the i.c.v. treatment, essentially using the methods described in previous studies [20, 21]. The SRT is based on the natural tendency of rats to actively investigate novel subjects while spending less time with familiar conspecific individuals. During a habituation session performed 24 h before the SRT, rats (n = 12 per group) were individually housed in transparent cages (48 × 37 × 21 cm) without food and water for 1 h. At the end of the habituation period, an unfamiliar juvenile (21–23-day-old male) was introduced into the cage for 4 min. At this age, juveniles are old enough to be considered individuals but not recognized by adults as territorial competitors and hence do not provoke potential aggressive activity [34].

On the test day, two trials and a control session were sequentially performed. During the first trial, an unfamiliar juvenile was introduced into the cage with the adult rat for 4 min. After an intertrial interval (30 min), the second test trial was conducted using the same, i.e., familiar, juvenile for a 4 min presentation to the adult rat. After another intertrial interval, the rat was exposed to a new, i.e., unfamiliar, juvenile for 4 min to verify the specificity of the memory. All trials were video recorded and the investigative behavior of the adult toward the juvenile, e.g., licking, sniffing, touching, chewing the fur of the juvenile and close following, was cumulatively calculated and recorded as the recognition ratio (RR): RR = T2/(T1 + T2), where T1 and T2 are the time (sec) spent investigating the juvenile rat during first and second sessions, respectively. Equal time spent by adults investigating a juvenile during the first and second sessions indicates no retention of social memory, and in this case, the RR value is 0.5. Significantly less time spent by an adult investigating a familiar juvenile during the second session indicates the retention of social memory, and in this case, the RR value will be lower than 0.5. Importantly, the intertrial interval is a critical parameter in this test and has been shown to be different for healthy rats (120 min) and for rats with AD-like pathology (30 min) [20, 21].

### Morris water maze (MWM)

The MWM test was performed on Day 29 after the i.c.v. treatment as described previously to assess short- and long-term spatial memory function [21]. Briefly, a 160 cm tank was divided into 4 quadrants, filled with water (temperature maintained at 21 °C) and surrounded by visual cues. A 10 cm escape platform was located in one of the quadrants and remained in place for all trials. Each rat (n = 12 per group) performed 3 consecutive trials per day for 3 days. Then, the platform was removed, and the rats were tested 24 h and 7 days after the last trial. At the start of each test, the rat was placed in one of the platform-free quadrants (the same order of the starting point was applied to all the rats on each day but was different between the trial days). The rat was allowed to swim for 90 s and was guided to the platform if it was unable to find the platform. Once the rat was on the platform, it remained in place for 20 s to provide orientation, after which it was handled for a 20 s interval period between trials. Trials were video recorded and analyzed with EthoVision software (Noldus, Wageningen, The Netherlands). The following dependent variables were recorded: latency to the platform, total swimming distance, swimming speed, time spent in the target quadrant (defined as the quadrant with the platform) and the number of crossings over the platform zone of 20 cm in diameter (defined as the zone surrounding the location where the platform was placed during the learning trials).

### Y-maze

In week five after the surgery, short-term memory was assessed using a Y-maze with arms symmetrically spaced at 120° angles. Each rat (n = 12 per group) was introduced into the same starting arm (arm B) and allowed to explore the maze freely for 10 min. The test takes advantage of the rat’s natural preference for novelty; hence, the rat explores the least recently visited arm. The placement of all four paws in the arm was scored as an arm entry. Recorded videos were analyzed to measure the total number of arm entries and percentage of spontaneous alternations (SAs). SAs were calculated as SA% = (Number of alternations/(Total number of arm entries − 2)) × 100. Pearson’s correlation of SA% to the number of arm entries was calculated to ensure an unbiased assessment of SA [35], but no correlation was observed (r = 0.2, *p* = 0.32).

### MRI-based measurement of brain volume

MRI was performed on a 9.4 T preclinical scanner (BioSpec 94/30 USR, Bruker Biospin, Ettlingen, Germany) interfaced with a Bruker AVANCE III console and controlled by Paravision 6.0.1 software (Bruker BioSpin). Imaging was performed with an 86 mm (ID) volume resonator and a 4-channel surface quadrature array receiver coil. We administered manganese as a contrast agent to increase the contrast in images of neuroanatomical structures, including hippocampal and brain laminar structures, which is known as manganese-enhanced MRI (MEMRI) [36, 37]. Rats (n = 6 per group) were intraperitoneally (i.p.) injected with 40 mg/kg MnCl_2_ 48 h and 24 h before scanning. During scanning, animals were anaesthetized with 1.5–2% isoflurane in a 1/1 mixture of air/oxygen. Respiratory signals and rectal temperature were monitored with a physiological monitoring system (SA Instruments, Inc., Stony Brook, NY, USA). A T2-weighted rapid acquisition with relaxation enhancement sequence [T2W-RARE; repetition time/echo time (TR/TE) = 8000/25 ms, rare factor = 4, number of averages (NA) = 2, field of view (FOV) = 34 × 25.6 mm, matrix size = 340 × 256, slice thickness = 300 µm, and acquisition time = 17 min] was acquired over the entire rat brain. The T2W-RARE images functioned as an anatomical reference that showed the overall structure of the brain and were used for the volume analysis. The image bias field created by the receiver coil was removed using Advanced Normalization Tools (ANTs N4 bias correction) [38], and bias field-corrected images were then skull-stripped using ITK-SNAP version 3.8.0 [39]. T2W-RARE images were first registered to a template image from the SIGMA rat brain atlas [40]. The volume of the whole brain and different areas of the EC and the hippocampus were measured as the number of voxels in the region of interest (ROI) and normalized to the total volume of the rat brain.

### Tissue collection

Rats (n = 6 per group, which were not used in the MRI scanning), were anesthetized by administering an i.p. injection of 0.6 ml of pentobarbital (200 mg/ml) mixed with lidocaine (20 mg/ml) and transcardially perfused with ice-cold 0.9% saline supplemented with 10 U/ml heparin (Heparin LEO, LEO pharma, Denmark). Dissected brains were split along the commissures, and the two hemispheres were sampled in an alternating manner to avoid lateralization. One entire brain hemisphere was postfixed with 4% paraformaldehyde for 48 h followed by embedding in paraffin, whereas the hippocampus from another hemisphere was quickly dissected, snap-frozen in liquid nitrogen, and stored at − 80 °C until further processing.

### Quantification of Aβ and pTau levels using ELISAs

Total proteins were extracted from frozen hippocampi (n = 6 per group) using N-PER (Neuronal Protein Extraction Reagent) supplemented with HALT protease inhibitor cocktail (both from Thermo Fisher Scientific) to measure the levels of Aβ species and pTau. Measurements of the total protein concentration, levels of Aβ species with the triplex Aβ Peptide Panel 1 (4G8) V-PLEX Kit and pTau levels with the pτ ELISA kit were performed as described above for human brain samples. Data from the Aβ and pTau assays were corrected for the total protein concentration.

### Stereology and immunohistochemistry (IHC)

A quantitative analysis of the total number of neurons in cornu ammonis 1 (CA1) and CA3 areas of the dorsal hippocampus (from − 1 mm to − 2.3 mm in the coronal plane relative to the bregma) from AD and control rats (n = 3 per group) was performed using systematic uniform random sampling (SURS) and optical disectors as described previously [21]. Every 8th 30 µm-thick section was deparaffinized and stained with modified Giemsa reagent. Stereological counting of cell numbers was performed with CAST-GRID software (Olympus, Denmark) using an Olympus BX50 microscope equipped with a camera.

For IHC, 7 µm-thick sections from AD and control rats (n = 6 per group, three coronal sections per animal) were deparaffinized, microwaved for 10 min in sodium citrate buffer (pH 6), washed with PBS and blocked with 5% bovine serum albumin (Sigma–Aldrich). Sections were incubated overnight with one of following primary antibodies: mouse anti-Aβ 6E10 (1:1000; Covance), rabbit anti-IBA1 (1:1000; WAKO), rabbit anti-GFAP (1:2000; DAKO), rabbit anti-cleaved caspase-3 (1:400; Asp175; Cell Signaling), mouse anti-synaptophysin 1 (anti-SYP1, 1:1000; SYSY) or mouse anti-VGLUT1 (1:250; SYSY). For staining with anti-Aβ 6E10, anti-IBA1 and anti-GFAP, sections were washed with PBS, and endogenous peroxidase activity was blocked with 3% H_2_O_2_ before sections were incubated with the corresponding secondary antibody (Envision HRP-labeled anti-mouse or anti-rabbit; DAKO). The immunoreaction was visualized using DAB, and the sections were dehydrated and coverslipped. For staining with anti-SYP1, anti-VGLUT1 and anti-cleaved caspase-3 antibodies, sections were incubated with secondary fluorescent antibody (goat anti-mouse conjugated to Alexa Fluor 568, 1:1000; Invitrogen), counterstained with a DNA dye (Hoechst 33258, 1:20000; Invitrogen) and coverslipped (ProLong Diamond Antifade Mountant; Invitrogen). For staining with the 6E10 antibody, sections were pretreated with 99% formic acid for 7 min before the heat-mediated antigen retrieval step. Brain sections from 28-month-old Tg Fischer 344-AD rats expressing mutated human *APP* and presenilin 1 (*PSEN1*) genes and non-Tg F344 WT control rats were analyzed using the protocol as positive and negative controls, respectively, to confirm the efficiency of the staining protocol for the 6E10 antibody.

### Image analysis

Bright field images of ROI (DG and CA) were recorded from IBA1 and GFAP labelled sections (10–15 images per ROI from three sections per animal, n = 6 per group) using a camera-equipped Olympus BX50 microscope coupled to CAST-GRID software (Olympus Denmark A/S, Ballerup, Denmark), ensuring systematic, uniform and random sampling. Images were adjusted to the same threshold level for each staining and the area of immunoreactivity was quantified as the average percentage of the image area occupied by positive immunoreactivity using PLAB Application software (version 0.3.6).

To quantify the integrated density, immunofluorescent images (SYP1, VGLUT1) were acquired using Zeiss Axio Observer microscope (Zeiss) with an Axiocam 702 camera coupled to ZEN image acquisition and analysis software using channels for DAPI and Alexa Fluor 568. ZEN Tiles was used to take ~ 60 images with a 20 × objective of the DG and CA1 region, then all images were stitched together to create one picture containing both regions. In total, two–three sections per rat (n = 6 rats per group) were included in the analysis. The images were prepared for analysis in ImageJ (version 1.53a) by delineating the DG and CA1 region followed by splitting the two channels. Converted grayscale images were used to measure mean gray value. To quantify cleaved caspase-3^+^-cells, 10–14 images per ROI (DG, CA and LEC) from three sections per animal (n = 6 per group) were recorded at × 20 magnification and data expressed as mean number of positive cells per square millimeter (cells/mm^2^).

For estimation of microglia morphology, three ROI (DG, CA, and LEC) from IBA1-labelled sections were imaged at × 20 magnification (2–3 images per ROI from each of three sections per animal, n = 6 animals per group). Changes in microglia ramified morphology were analysed using the ImageJ software and an adapted version of previously described protocol [41]. Briefly, each image was binarized, then FFT Bandpass filter was applied followed by the consisted threshold settings application. The unsharp mask and despeckle tools were also used to refine the image. Then the image was converted to a skeleton form and Analyze Skeleton plugin was applied to acquire the summed branch number (endpoints) and junction number per soma count per image frame using cut off parameters as previously described [41].

### Gene expression analysis

Total RNA was isolated from the hippocampi of AD rats and control rats (n = 6 per group) using an RNeasy Lipid Tissue Mini Kit and subjected to DNase on-column digestion (both from Qiagen). Next, cDNAs were synthetized using a High-Capacity cDNA Reverse Transcription Kit (Thermo Fisher, Waltham, MA, USA) according to the manufacturer’s instructions. qPCR was performed with Fast SYBR Green Master Mix (Applied Biosystems) and a Stratagene Mx3005p qPCR System (Agilent Technologies). C_t_ values were normalized to the expression of two reference genes, *ACTB* and *RPL13a*, using the geometric mean. The primers for qPCR (TAG Copenhagen) were designed using Primer-BLAST and tested by Oligo Primer Analyses (version 7); the sequences are listed in Additional file 6: Table S2.

### Statistical analysis

Data obtained from human samples, behavioral experiments, MRI, histology, ELISAs and qPCR were analyzed using the *t*-test or Mann–Whitney *U* test with GraphPad Prism version 7.00 software (GraphPad, San Diego, CA, USA) to determine the significance of differences between groups. The D’Agostino-Pearson omnibus normality test was used to identify whether the data followed a Gaussian distribution. One-sample Student’s *t*-tests were performed to estimate whether datasets were greater than the chance level (reference value of 0.5 for the SRT). All numerical results are presented as the means ± SEM. *P* values < 0.05 were considered statistically significant, and levels of statistical significance are denoted as follows: *p < 0.05, **p < 0.01 and ***p < 0.001.

## Results

### Preparation of AD brain tissue extracts enriched with soluble Aβ peptides and AD-related proteins

**Fig. 1:**
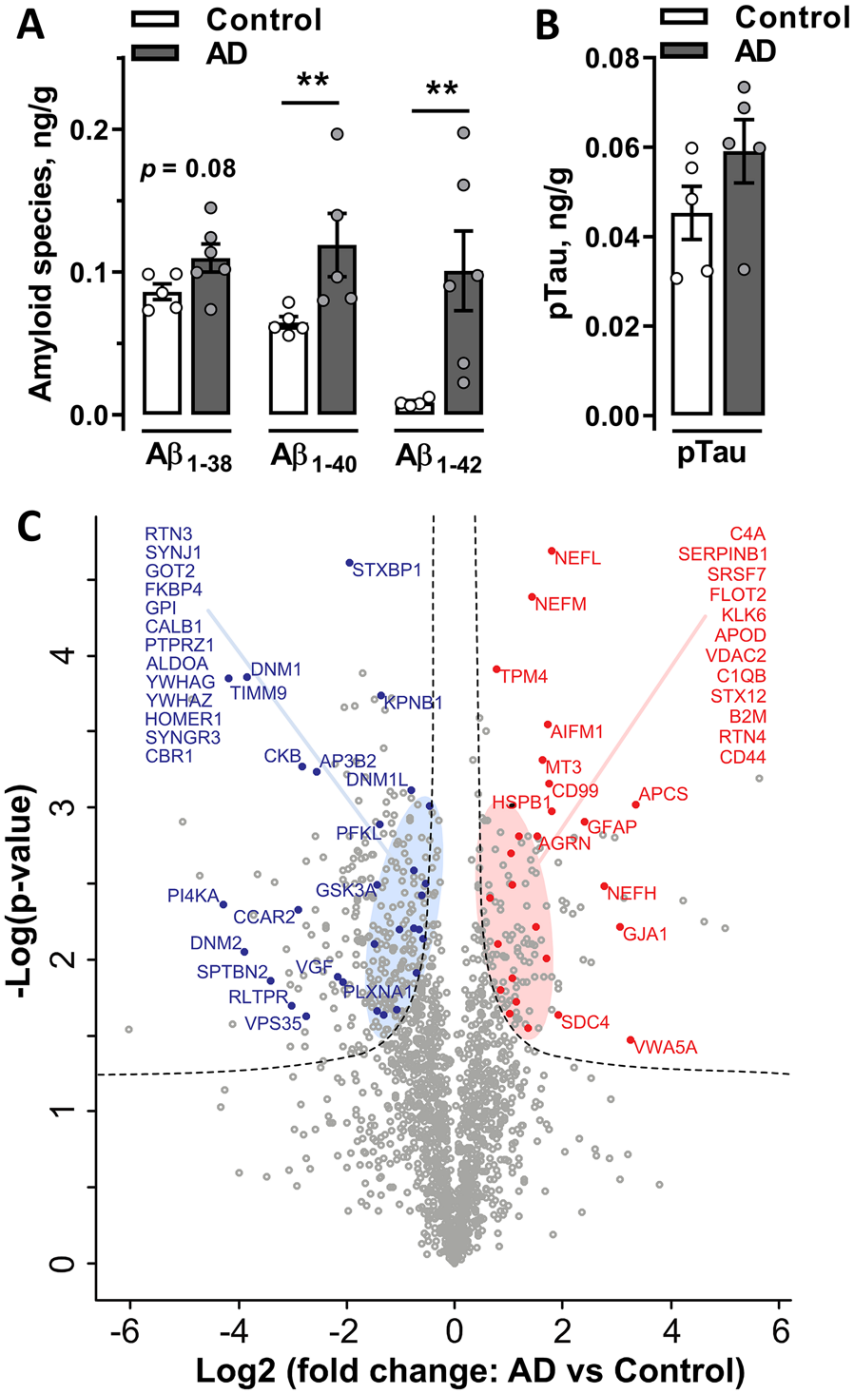
Characterization of water-soluble proteins eluted from AD and non-AD human brain tissues. **A** The eluted soluble fraction from human brains were enriched in Aβ_1–40_ and Aβ_1–42_ and showed a tendency toward Aβ_1–38_ enrichment in individual AD extracts (n = 6) compared to non-AD control samples (n = 5). **p < 0.01. **B** The levels of pTau were not significantly different between AD and control protein extracts (n = 5 per group). Data are presented as the means ± SEM. **C** Volcano plot visualizing up- and downregulated proteins (FDR < 0.01 and s0 = 0.1). The plot shows − log10 transformed *p* values versus log2-transformed fold changes in mean protein intensities between AD and control pooled extracts. Selected functionally relevant proteins are highlighted in the plot, showing less abundant (left panel, blue) or more abundant (right panel, red) proteins in AD extracts compared to non-AD control extracts

Soluble protein extracts were derived from human brain tissues with neuropathologically validated AD and age-matched non-AD control samples (Additional file 5: Table S1). Individual AD and non-AD samples were analyzed using ELISA to confirm that prepared AD extracts were enriched with soluble Aβ peptides. Significantly higher levels of soluble Aβ_1–40_ (*p* = 0.043) and Aβ_1–42_ (*p* = 0.030) peptides were detected in AD compared to control brain extracts, while a trend toward statistical significance was observed for the level of the Aβ_1–38_ peptide (*p* = 0.080; Fig. 1A). Correspondingly, pooled AD samples have higher levels of Aβ_1–40_ (115.4 pg/ml) and Aβ_1–42_ (36.5 pg/ml) compared to non-AD pooled control (76.8 pg/ml and 12 pg/ml, respectively). No significant difference in the level of soluble pTau was measured between the AD and non-AD individual extracts (*p* = 0.174; Fig. 1B) and between pooled samples.

Native AβΟs are considered key mediators of synaptic toxicity [8]. However, additional proteins might be eluted together with AβOs, which, in turn, may enhance the activity of these toxic oligomers. Therefore, we next performed an unbiased in-depth investigation of the protein profiles of AD and control brain pooled extracts using quantitative mass spectrometry-based proteomics. In total, 1750 proteins were identified after filtering. Complete lists of the identified proteins are provided in Additional file 7: Table S3. Among all proteins detected, 405 proteins were differentially expressed between AD and control samples, with 131 proteins more abundant in the AD extracts and 274 less abundant in AD extracts compared to control extracts (Fig. 1C). Many of the proteins identified as more abundant in the AD group have previously been associated with AD pathology, whereas some coeluted proteins might potentially alter the toxicity of AβOs.

### Central treatment with native AβOs decreased social memory but did not affect spatial memory

**Fig. 2:**
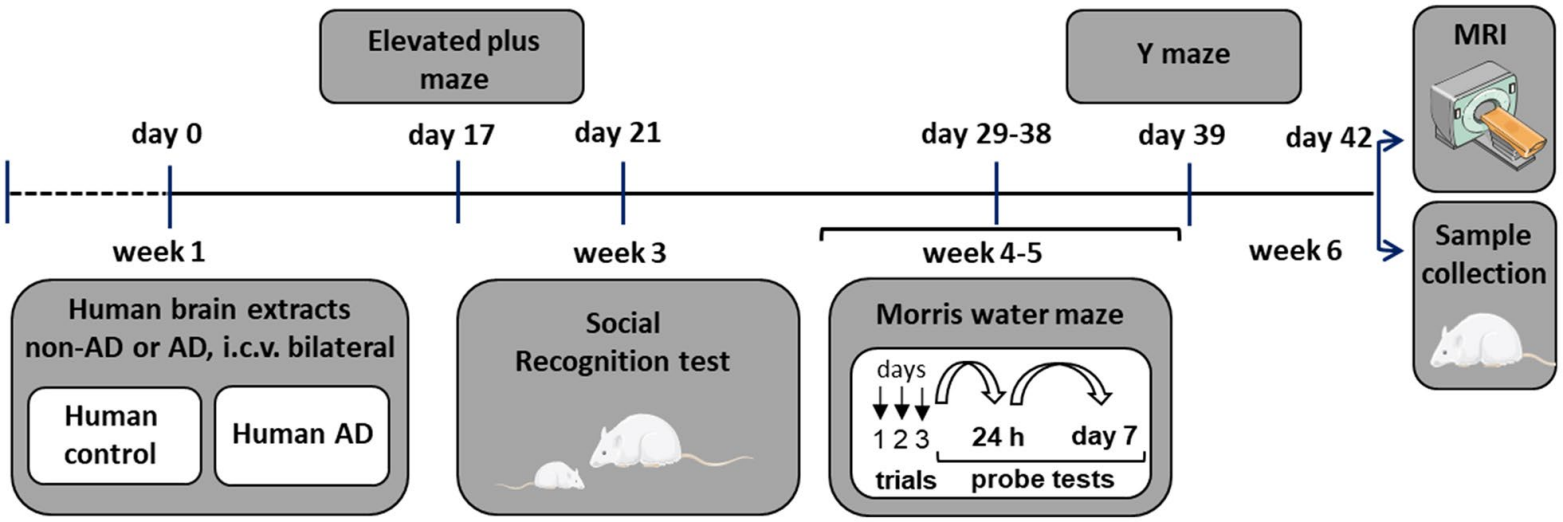
Timeline of the experimental protocol. Rats were i.c.v. injected with AD or non-AD human brain tissue extracts and, after a recovery period, subjected to a battery of behavioral tests and scanned with MRI before sample collection

**Fig. 3:**
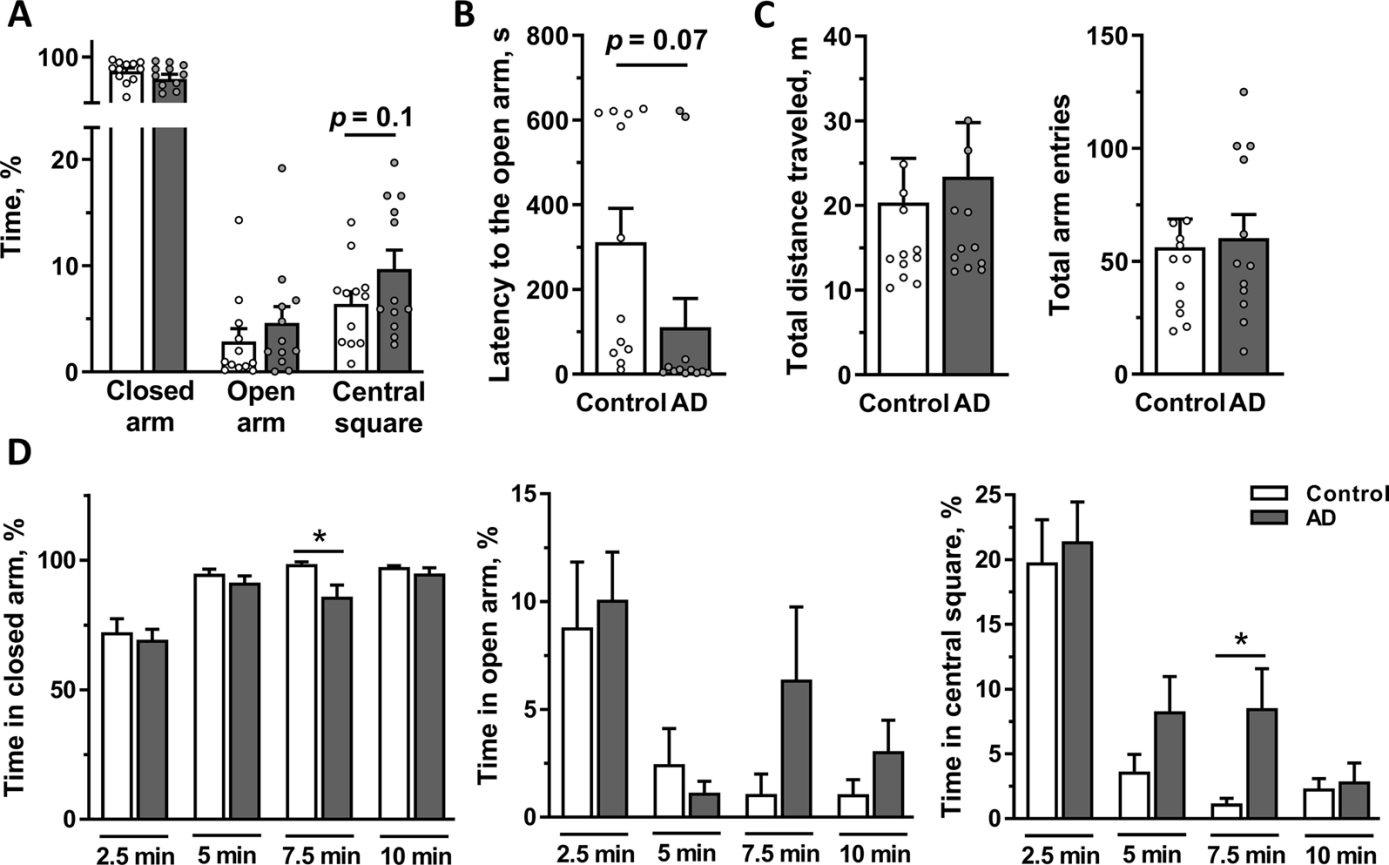
Anxiety-related behaviors assessed using the EPM test. Rats were i.c.v. injected with AD or non-AD control extracts (n = 12 per group) and tested in the EPM for 10 min on Day 17 postinjection. **A** Time spent in the closed and open arms and in the central square expressed as percentage of total time. **B** Latency to the first entry into an open arm. **C** Total distance traveled and total number of arm entries were similar between the AD and control groups. **D** Percentage of time spent in the closed and open arms and the central square analyzed for each 2.5 min time interval. Data are presented as the means ± SEM. *p < 0.05

Prepared brain tissue extracts were bilaterally i.c.v. injected into adult male rats, and the animals were subjected to a battery of behavioral tests after a postsurgery recovery period of 2.5 weeks (Fig. 2). First, we analyzed anxiety using the EPM. Regardless of treatment, rats spent most time in the closed arms due to an innate fear of height, although they also exhibited exploratory behavior by venturing out of the closed arms into the center (relative time spent, 2.9 ± 1.2% for the control group and 4.6 ± 1.6% for the AD group) or into the open arms (6.3 ± 1.2% for the control group and 9.7 ± 1.9% for the AD group). AD rats showed a trend toward an increased percentage of time spent in the central square (*p* = 0.10) and lower latency to the first entrance into the open arm (*p* = 0.07) compared to the control group, without reaching statistical significance (Fig. 3A, B). Interestingly, when the recorded video (10 min) was analyzed for each quarter of the test (2.5 min), AD rats spent a significantly less percentage of time in the closed arms and a greater percentage of time in the central square during the 3rd quarter of the recorded period (*p* = 0.02 and *p* = 0.02, respectively; Fig. 3D, left and right panels), suggesting a temporary anxiolytic-like effect on the AD group. A similar effect was observed for the number of entries into the central square (*p* = 0.03; Additional file 1: Fig. S1A). Rats in both groups traveled a similar total distance and had a similar number of arm entries, indicating that locomotor activity was not different between groups (Fig. 3C).

**Fig. 4:**
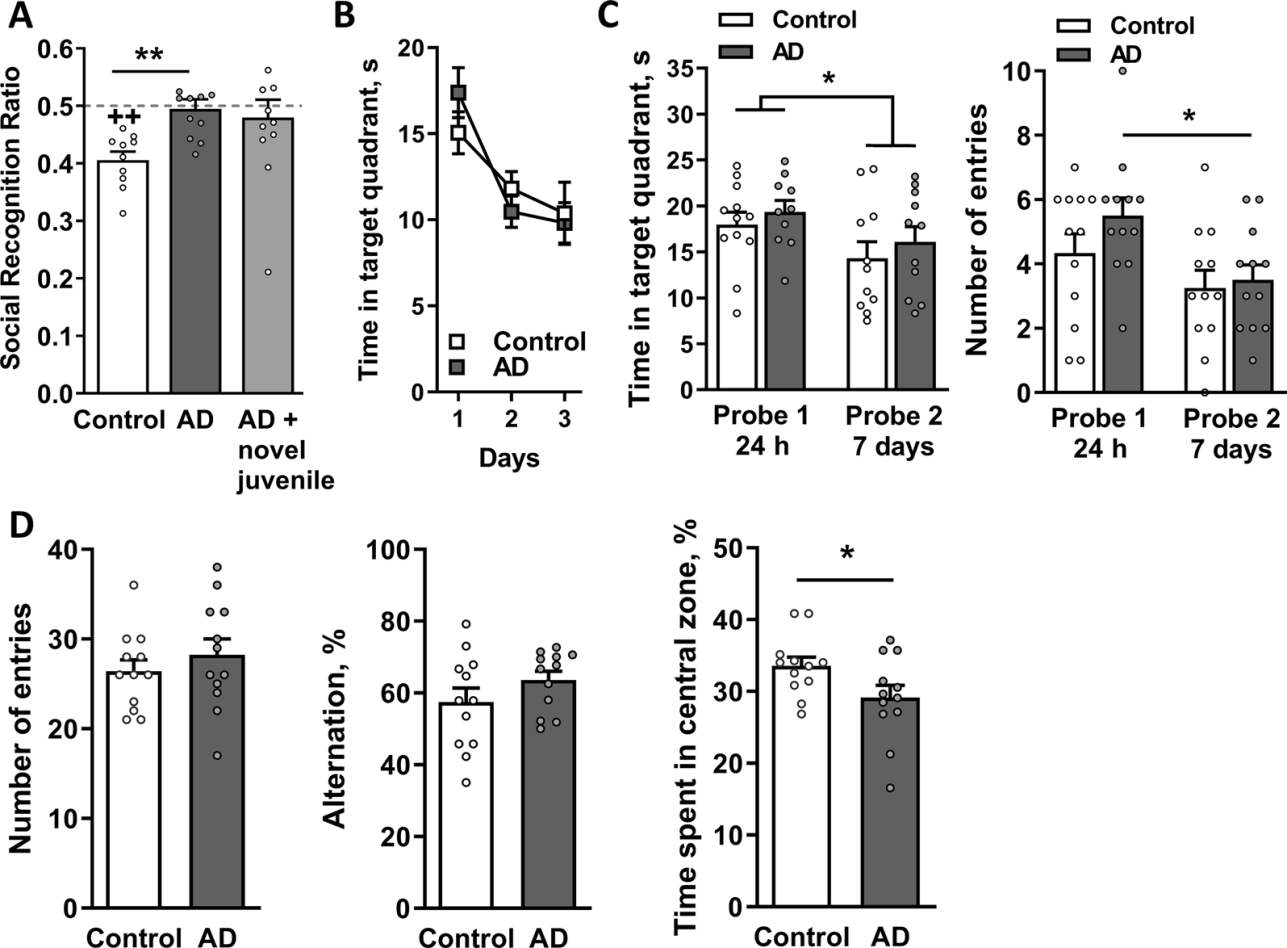
AD rats have a deficit in the retention of social memory but have no short- or long-term spatial memory deficits. Rats were i.c.v. injected with AD or non-AD control extracts (n = 12 per group) and tested for **A** social memory on Day 21 after the infusion. Data were analyzed using Student’s *t*-test to compare social recognition ratios (RRs) between groups and one sample *t*-test to compare each group to a chance value of 0.5. Data are presented as the means ± SEM. **p < 0.01 compared with control rats,  ++*p* < 0.01 compared to the hypothetical chance value of 0.5 (dashed line). **B** Control and AD rats started acquisition training in the MWM on Day 29 after the i.c.v. infusion, and **C** reference memory was tested 24 h and 7 days after the last training session. During the 3 days of acquisition training, the time spent in the target quadrant decreased, indicating spatial learning and memory formation in both groups. (**C**, left panel) Rats from both groups spent significantly less time in the target quadrant and (**C**, right panel) made fewer entries into the platform zone during the second probe trial (Day 7) compared to the first probe trial (24 h). Data were analyzed using two-way-ANOVA, *p < 0.05. **D** The Y-maze test, which was performed on Day 39 postinjection, revealed no difference in either the number of entries (**D**, left panel) or the percentage of alternations (**D**, center panel) between the AD and control groups. (**D**, right panel) The AD rats spent a significantly lower percentage of time spent in the central zone of the Y-maze than control rats. Data were analyzed using Student’s *t*-test, *p < 0.05. **A**–**D** n = 12 rats per group

At three weeks postinjection, rats were tested for the retention of social memory. Rats from both groups spent similar amounts of time investigating the juvenile during the first presentation trial, indicating that the injection per se had no effect on investigation activity (*p* = 0.32; data not shown). During the second trial, control rats explored the familiar juvenile for a shorter period, as reflected by a significant reduction in the RR value compared to the theoretical value of 0.5 (*p* = 0.002), indicating the presence of social discrimination memory (Fig. 4A). However, AD rats exhibited a significant deficit in social memory; the RR value of the AD group (0.49 ± 0.02) did not differ from 0.5 (*p* = 0.6) and was significantly different from that of the control group (*p* = 0.001 by two-tailed *t*-test; Fig. 4A). Rats were exposed to a novel juvenile once again to exclude potential nonmnemonic effects. The AD rats exhibited a normal investigation behavior toward the new unfamiliar juvenile in this subsequent session (*p* > 0.05; Fig. 4A), similar to the control group (data not shown).

Spatial memory was analyzed using the MWM test at week four postinjection. During training trials, rats in the AD and control groups equally learned the platform location, showing similar time spent in the target quadrant (Fig. 4B) and latency to the platform (Additional file 1: Fig. S1B, left panel). In the probe trials, AD rats also performed similarly to the control group in both the 24-h and 7-day trials (Fig. 4C and Additional file 1: Fig. S1B, right panel). When comparing the two probe trials, AD rats had significantly fewer entries into the platform zone on Day 7 (*p* = 0.03), indicating a reduction in memory retrieval (Fig. 4C, right panel), whereas the control group did not display this tendency. The total distance traveled during the training and probe trials was similar between groups, indicating no motor deficits in this animal model (Additional file 1: Fig. S1C).

AD rats placed in the Y-maze did not differ from the control group in the number of entries, thus showing similar locomotor activity (*p* = 0.4; Fig. 4D, left panel). Neither group exhibited a difference in short-term spatial working memory, as reflected by fairly similar levels of SAs (*p* = 0.2; Fig. 4D, central panel). Rats from both groups spent approximately the same time in all arms (*p* = 0.48, data not shown), whereas AD rats spent significantly less time in the central zone (*p* = 0.047; Fig. 4D, right panel).

Overall, these results indicate that AD rats have unaffected spatial memory and anxiety levels and motor skills, while performance in the SRT was disturbed within 6 weeks postinjection.

### Loss of volume in the LEC after the i.c.v. injection of human AD extracts

**Fig. 5:**
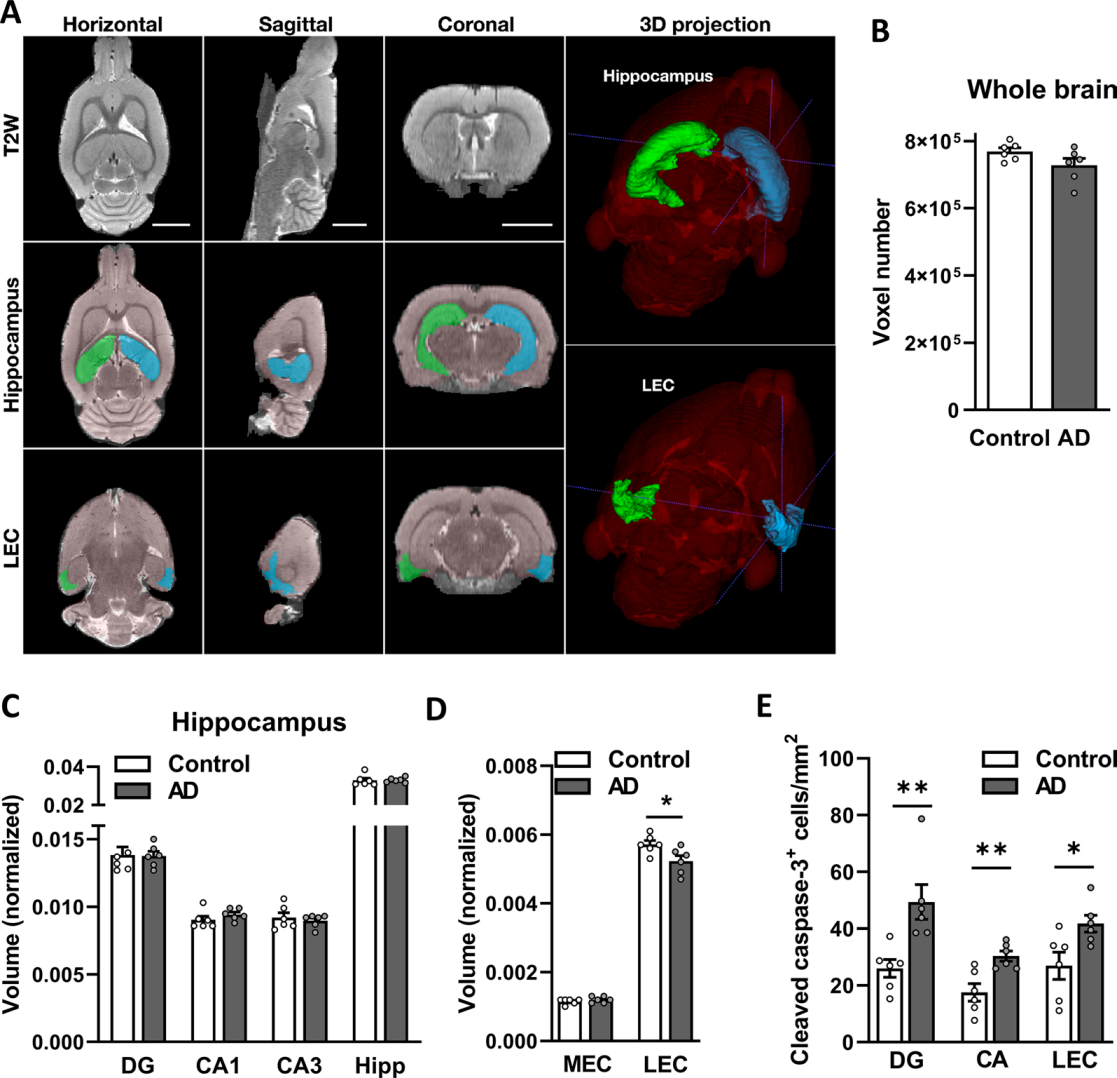
Measurements of the volume of different brain regions using MRI. Rats were scanned using MRI (n = 6 per group) to measure the volumes of different brain regions as the number of voxels in the ROI. **A** Representative horizontal, sagittal, and coronal T2-weighted RARE images are shown. 3D-reconstructed images were registered and segmented with a template image from the SIGMA rat brain atlas. The scale bar in each panel represents 5 mm. Representative ROIs used in volume measurements, such as hippocampi and LEC (blue—right; green—left), are highlighted on the three orthogonal slices and the 3D projection. Volume was measured in **B** the whole brain, **C** regions of the hippocampus and **D** regions of the EC. Data from the EC and the hippocampus were normalized to total brain volume. Data are presented as the means ± SEM. *p < 0.05. DG: dentate gyrus. *CA* cornu ammonis, *MEC* medial entorhinal cortex, *LEC* lateral entorhinal cortex, *EC* entorhinal cortex

Volumetric and histopathological analyses of the animals treated with AD or control brain extracts were performed at the end of week 6 postinjection using a separate set of animals for each type of analysis (Fig. 2). MRI volume measurements of the whole brain displayed no statistically significant difference between the AD and control groups, although a tendency toward a decrease in volume was observed in the AD group (*p* = 0.1; Fig. 5B). The volumes of the entire hippocampus and its different subregions were quantified and normalized to the total brain volume (Fig. 5A). Compared to control rats, no significant volume loss was measured in the dentate gyrus (DG) (*p* = 0.92), CA1 (*p* = 0.12) and CA3 (*p* = 0.97) of AD rats, and no statistically significant differences in the volume measurements of the entire hippocampus were recorded (*p* = 0.4; Fig. 5C). Using the MEMRI approach, the voxel intensities of ROIs were compared between the AD and control groups. No significant differences were identified in any of the investigated areas (Additional file 2: Fig. S2A–C). These findings were substantiated in a separate group of animals by stereological quantification of neurons in the CA1 and CA3 regions of the dorsal hippocampus, where no statistically significant differences in the total number of neurons were observed between the AD and control groups in the CA1 (*p* = 0.59) and CA3 (*p* = 0.33) regions of the hippocampus (Additional file 2: Fig. S2D). The EC, another brain region sensitive to AD pathology [42–44], is anatomically connected to the hippocampus and subdivided into two regions: the lateral entorhinal cortex (LEC, Fig. 5A; Additional file 3: Fig. S3A) and medial entorhinal cortex (MEC). Measurements of the LEC volume revealed a statistically significant decrease in the AD rats (*p* = 0.03; Fig. 5D) but no difference in the MEC (*p* = 0.53) or entire EC (*p* = 0.26). Furthermore, number of cleaved caspase-3^+^ cells were significantly higher in the DG, CA and LEC of AD rats compared to control (Fig. 5E). A trend toward a negative correlation between the levels of social memory (RR index) and total brain volume (*p* = 0.07, r =  − 0.57) and between the RR index and LEC volume (*p* = 0.08, r =  − 0.55) was observed (Additional file 2: Fig. S2E–F).

In summary, the MRI analysis revealed a decrease in the volume of the LEC in AD rats, associated with cellular death, while no other analyzed areas displayed a volume loss.

### Upregulation of early-stage AD-related genes in the hippocampus of AD rats in the absence of plaques

**Fig. 6:**
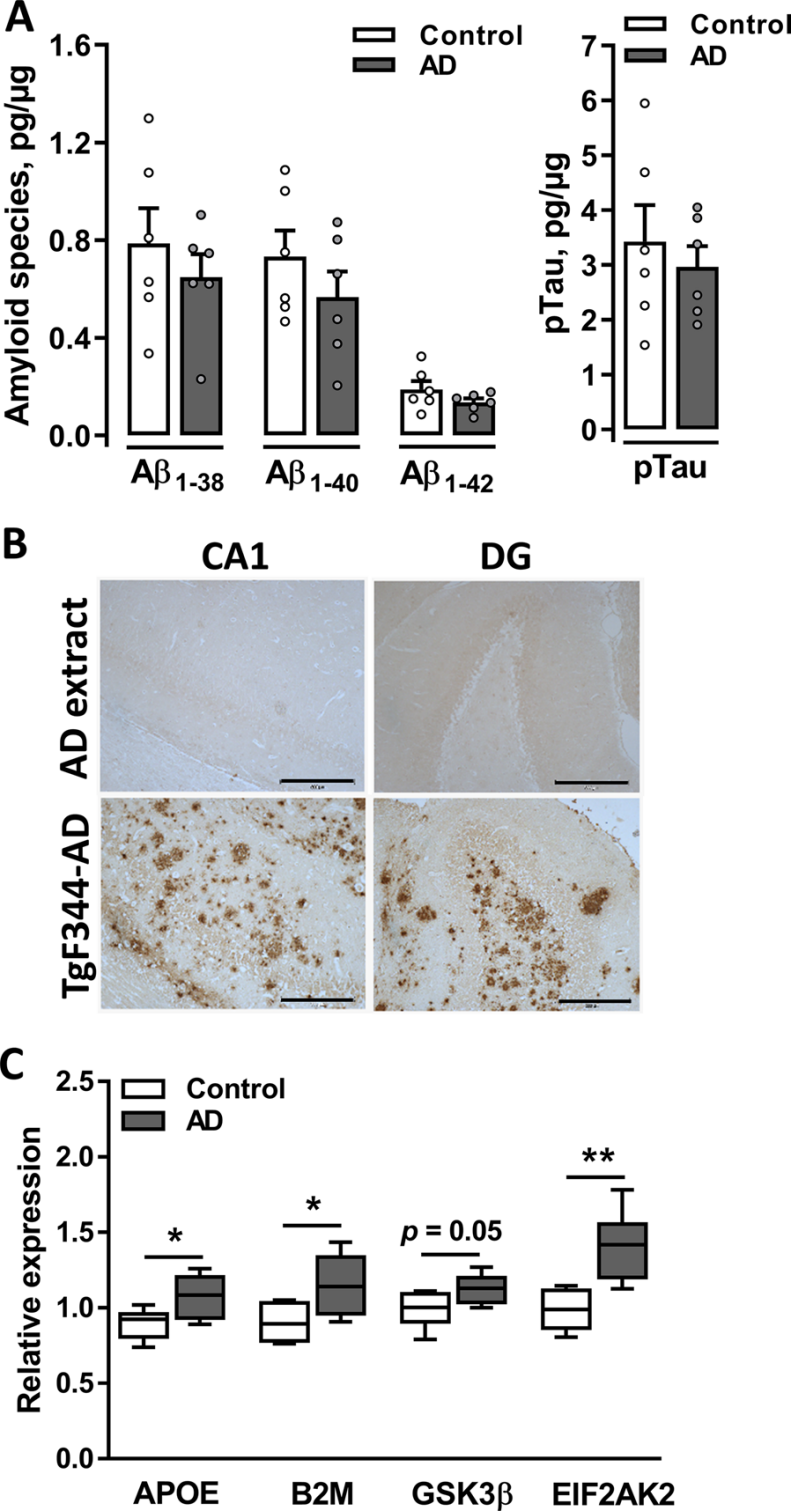
Molecular alterations in the rat brain following the AβO infusion. **A** Quantification of the levels of amyloid peptides (left panel) and pTau (right panel) in rat hippocampal tissues from AD and control animals (n = 6 per group) using ELISAs. **B** IHC with the 6E10 antibody showed no plaques in the brains of AD rats. The same antibody strongly stained Aβ plaques in the brains of 28-month-old TgF344-AD rat, which were used as a positive control. Representative images of CA1 and DG regions of WT rats injected with AD human brain tissue extract (top panels) and Tg rats (bottom panels) are shown. Scale bar, 300 µm. **C** The relative mRNA expression of early AD markers in AD and control animals (n = 6 per group) measured using qPCR. Data were normalized to two reference genes: *ACTB* and *RPL13A*. Data are presented as the means ± SEM. *p < 0.05, **p < 0.01

The hippocampal abundance of soluble Aβ species (Aβ_1–38_, Aβ_1–40_ and Aβ_1–42_) and pTau were measured using ELISAs to directly assess the effect of bilateral injections of native AβO extracts on potential amyloid pathology. No statistically significant differences were observed in the levels of Aβ peptides and pTau between the AD and control rats (Fig. 6A). Furthermore, IHC staining with the anti-Aβ 6E10 antibody did not detect any signs of Aβ plaques in the hippocampus or other brain regions in the AD and control rats (Fig. 6B, top panels, and Additional file 3: Fig. S3B, respectively). Brain sections from 28-month-old F344 WT and TgF344-AD rats expressing human mutated *APP* and *PSEN1* genes were analyzed to evaluate the efficiency and specificity of the antibody [45]. As expected, the WT rats showed no detectable Aβ plaques (Additional file 3: Fig. S3B, bottom panels), while the TgF344-AD rats, which were used in this study as a technical positive control, displayed multiple Aβ plaques in the hippocampal area (Fig. 6B, bottom panels), thereby confirming the efficiency and specificity of the anti-Aβ antibody and the staining protocol.

The progression of AD from early to late stages was recently described at the level of gene expression [46, 47]. RT–qPCR was performed to assess the mRNA expression of selected early and late markers of AD. Several early-stage AD-related markers were significantly upregulated in the hippocampus of the AD rats, such as *APOE* (apolipoprotein E; *p* = 0.028), *EIF2AK2* (eukaryotic translation initiation factor 2 α kinase 2; *p* = 0.003) and *B2M* (β-2-microglobulin; *p* = 0.037), together with the finding of a trend toward the upregulation of the tau-phosphorylating enzyme *GSK3β* (glycogen synthase kinase 3 β; *p* = 0.052; Fig. 6C). However, the expression of genes described as markers of late AD stages [47] was unaffected: *TREM2* (triggering receptor expressed on myeloid cells 2; *p* = 0.197), *LPL* (lipoprotein lipase; *p* = 0.485) and *CST7* (cystatin F; *p* = 0.240) (Additional file 4: Fig. S4).

Together, these data indicate that central treatment with native AβOs resulted in increased mRNA expression of multiple early AD-related markers 6 weeks after i.c.v. infusion in the absence of Aβ deposits and detectable levels of soluble Aβ species and pTau.

### Bilateral injection of native human AβOs induced prolonged neuroinflammation and synaptic reorganization in the hippocampus

**Fig. 7:**
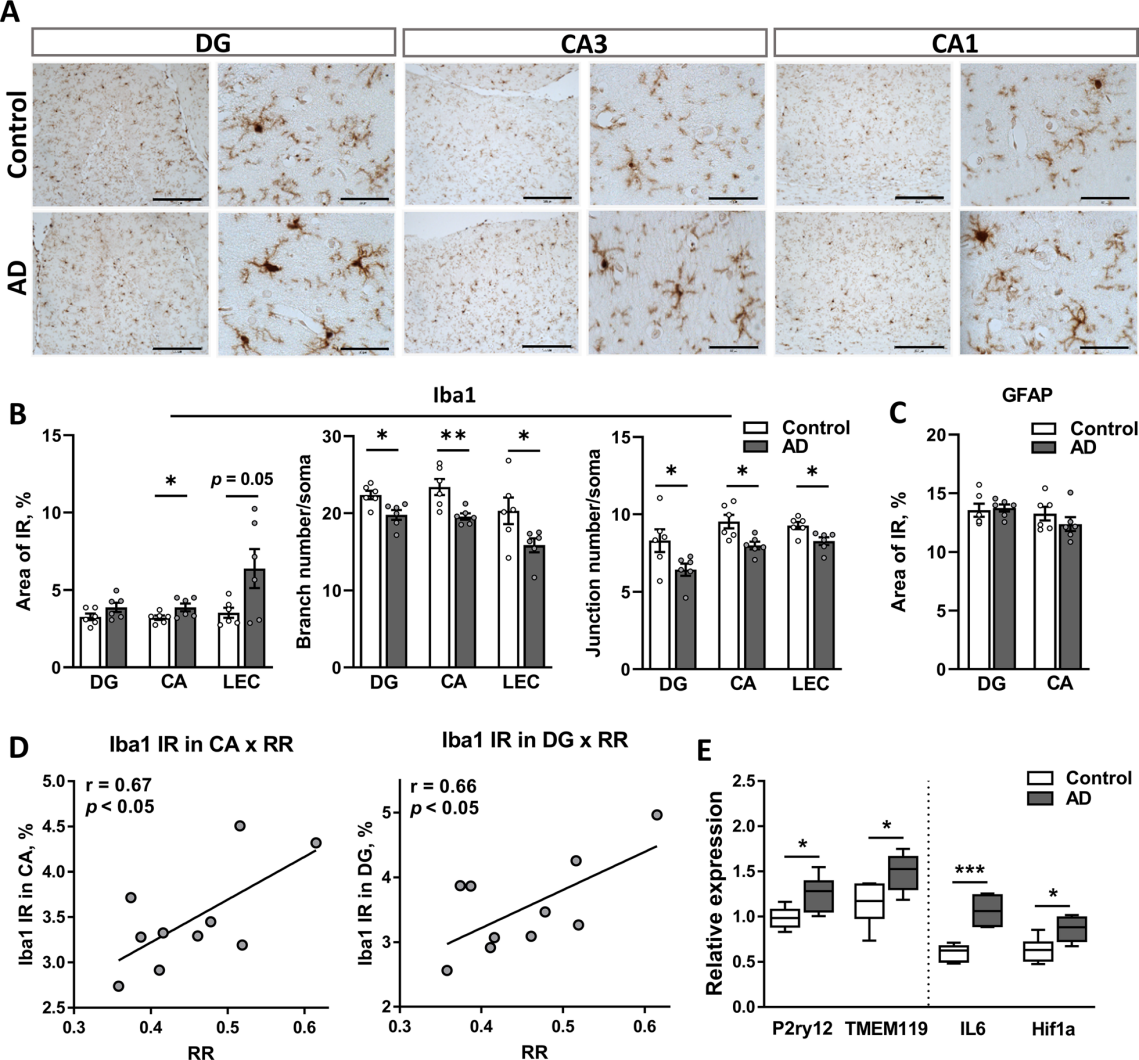
The i.c.v. injection of AβOs induces brain inflammation. **A** Representative images of IBA-positive microglial cells at low and high resolutions (scale bar 300 and 50 µm, respectively) in the hippocampal CA1, CA3 and DG showing higher IR in the AD group. **B** Analysis of microglia altered morphology. Left panel: quantification of the IBA1-positive area in the DG and CA regions of the hippocampus and LEC. The number of branches (middle panel) and junctions (right panel) in microglial cell process per IBA1-positive cell was quantified in three regions (DG, CA and LEC). **C** Quantification of the GFAP-positive area in the CA and DG regions of the hippocampus. **D** Pearson’s correlation analysis indicates a strong positive correlation between the RR index and the level of IBA1-IR in the CA (E, left panel) and DG (E, right panel). **E** The relative mRNA expression of multiple inflammatory markers measured using qPCR. Data were normalized to two reference genes: *ACTB* and *RPL13A*. Data are presented as the means ± SEM. **B**–**C** and **D** n = 6 rats per group. *p < 0.05, ***p < 0.001. *AD* Alzheimer’s disease, *DG* dentate gyrus, *CA* cornu ammonis, *IBA1* ionized calcium binding adaptor molecule 1, *P2RY12* purinergic receptor P2Y12, *TMEM119* transmembrane protein 119, *IL6* interleukin 6, *HIF1α* hypoxia-inducible factor 1 α

Neuroinflammation is substantially involved in the pathogenesis of AD [48, 49]. Rat brain tissue was labeled with anti-IBA1-(ionized calcium binding adapter molecule 1) and anti-GFAP-specific (glial fibrillary acidic protein) antibodies to examine microgliosis and reactive astrogliosis in the hippocampus, respectively, and to evaluate a possible cerebral inflammatory response in this model. The analysis of the levels of immunoreactivity (IR) for IBA1, a homeostatic microglial protein essential for microglial migration and phagocytosis, showed an increase in the CA (*p* = 0.043), with a similar trend in the DG (*p* = 0.124) and LEC (*p* = 0.05) of AD rats compared to controls (Fig. 7A, B). Furthermore, number of branches or junctions per IBA1^+^ soma were significantly lower in AD group (Fig. 7B, middle and right panels), indicating the reactive microglia morphology. No difference was observed in GFAP-IR in either the CA or DG between groups (Fig. 7C), indicating that microglial activation, but not astrogliosis, is present 6 weeks after the i.c.v. injection of AβOs. Pearson’s correlation analysis showed a strong positive correlation between the RR index and the level of IBA1-IR in both the CA (r = 0.67, *p* = 0.03) and DG (r = 0.66, *p* = 0.04), indicating an association between a decline in short-term social memory and hippocampal neuroinflammation (Fig. 7D).

Based on the microglial proinflammatory response observed at the histological level, we next investigated the expression of genes encoding the inflammation-related markers *IL6* (interleukin 6), *IL1β*, *TNFα* and oxidative stress-related transcription factor *HIF1a* (hypoxia inducible factor 1α). In addition, the expression of two putative homeostatic microglial markers, *P2RY12* (purinergic receptor P2Y12) and *TMEM119* (transmembrane protein 119) [50], in the hippocampus of AD and control rats was evaluated. The expression of these four genes was significantly upregulated in the AD group compared to the control group, confirming the neuroinflammatory response in AD rats (Fig. 7E). The expression of acute inflammatory genes IL1β (*p* = 0.999) and TNF*α* (*p* = 0.859) was unaffected (Additional file 4: Fig. S4).

**Fig. 8:**
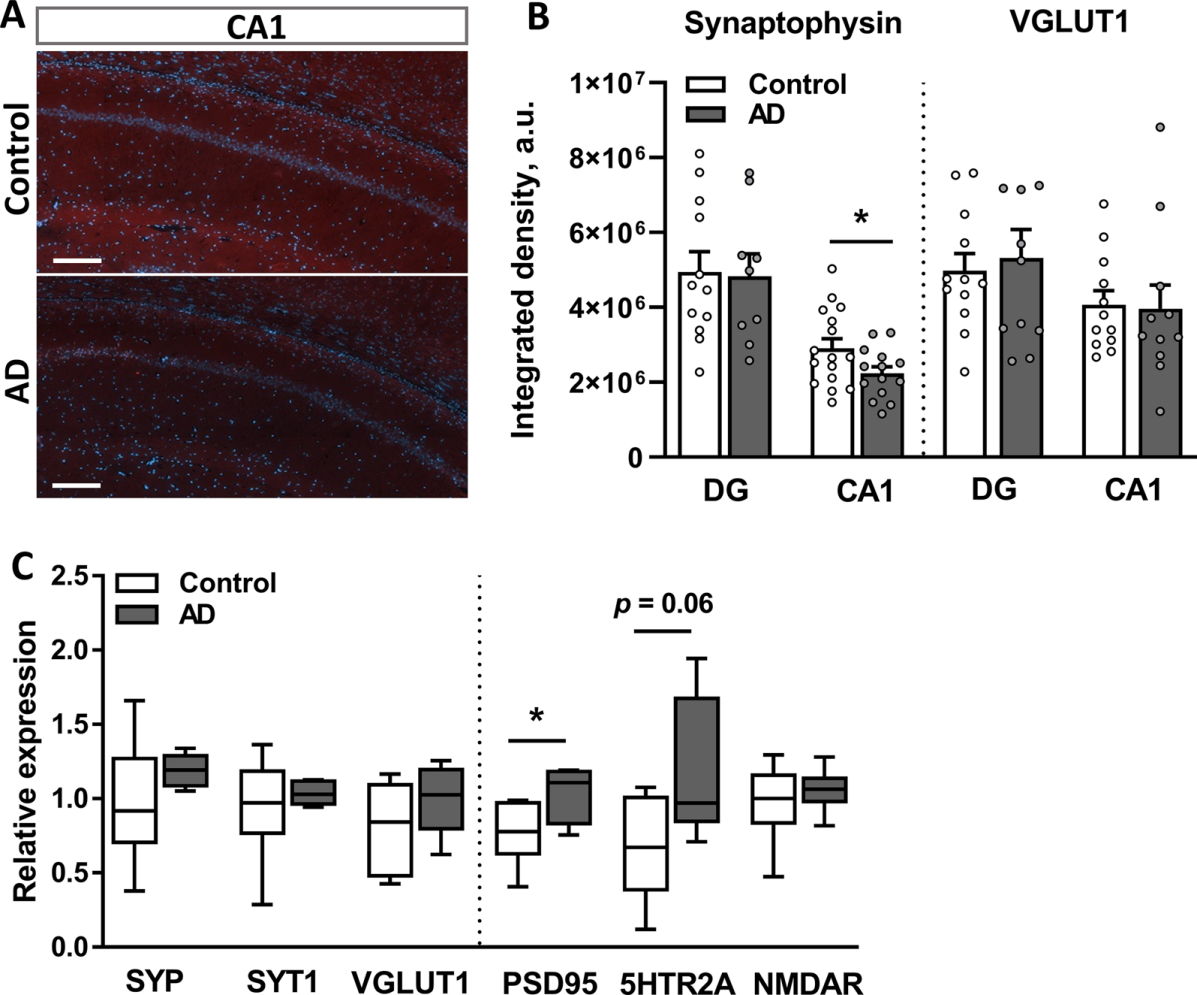
The i.c.v. injection of AβOs alters synaptic plasticity in the hippocampus. **A** Representative images of immunofluorescence staining for synaptophysin within the CA1 and DG of AD and control rats. Scale bar, 200 µm. **B** Quantification of synaptophysin- and VGLUT1-positive IR areas in the DG and CA1 regions of the hippocampus, which are reported as integrated densities. Data are from two–three sections per rat, 6 rats per group. **C** The relative mRNA expression of synaptic markers measured using qPCR. Data were normalized to two reference genes: *ACTB* and *RPL13A*. **B**–**C** Data are presented as the means ± SEM. *p < 0.05. n = 6 rats per group

Soluble AβOs are known to be synaptotoxic both in vitro and in vivo [14, 23]. Therefore, we next addressed the number of synaptic terminals in the hippocampus of AD and control rats. Indeed, IHC analysis revealed a significant decrease in synaptophysin-IR (SYP-IR) in the CA1 area (*p* = 0.048) but not in the DG of the AD group (*p* = 0.426; Fig. 8A, B, left panel). However, the number of glutamatergic terminals, which was estimated based on vesicular glutamate transporter 1 (VGLUT1)-IR, was unaffected in the CA1 (*p* = 0.54) and DG (*p* = 0.60) of the AD rats compared to control rats (Fig. 8B, right panel). The expression levels of six genes encoding synaptic proteins, such as *SYP*, synaptotagmin-1 (*SYT1*), *VGLUT1*, postsynaptic density protein 95 (*PSD95*), serotonin 2A receptor (*5HTR2A*) and N-methyl-D-aspartate receptor (*NMDAR*) (all previously described to be involved in AD-related synaptic dysfunction [14, 51–53]) were analyzed using RT–qPCR and showed significant upregulation of *PSD95* (*p* = 0.044) and a tendency toward *5HTR2A* upregulation (*p* = 0.06) in the AD group (Fig. 8C).

Together, these results suggest the induction of a prolonged neuroinflammatory response mediated by microglia along with hippocampal region-dependent synaptic remodeling in response to the injection of the AD brain tissue extract.

## Discussion

Here, we characterized cognitive functions and structural and molecular alterations in the brains of rats, focusing on early AD-related pathological changes after a bilateral i.c.v. infusion of native soluble AβOs from human patients with AD and compared them with non-AD-treated controls. AD rats showed significantly decreased social memory, while spatial-related cognitive functions and anxiety were unaffected within 6 weeks after injection. We performed structural and biochemical characterization of brains at the end of the in vivo experiments, i.e., 6 weeks after the infusion, to complement the behavioral findings. Despite unaltered tissue levels of soluble AβΟ peptides, the plaque-free hippocampus and entorhinal cortex of AβO-injected rats showed signs of neuroinflammation, as reflected by local microgliosis and altered expression of inflammation-related genes typical of early stages of AD. These changes were accompanied by an increase in number of cleaved Caspase-3^+^-cells, reduction in the LEC volume and a tendency toward a decrease in the whole brain volume, as confirmed by MRI.

Multiple lines of evidence have suggested that soluble AβOs are the driving factors for AD pathological changes since brain extracts depleted of these species are unable to induce β-amyloidosis in Tg mice, alter synaptic plasticity, inhibit hippocampal LTP in vivo or ex vivo in hippocampal slices and are not neuro- or synaptotoxic [12–14, 54–57]. A small fraction of native soluble AβOs, representing less than 0.05% of total brain tissue-extracted amyloid, has previously shown a strong ability to induce amyloid seed formation in young APP Tg mice [58] and was approximately 100-fold more potent than synthetic Aβ peptides in terms of neuritotoxicity and seeding activity [12, 57, 59]. However, additional cofactors were suggested to influence these processes [12]. Here, we characterized the protein profiles of extracts eluted from human AD cortical samples and compared them with age-matched non-AD controls. In addition to the expected increases in levels of Aβ_1–40_ and Aβ_1–42_ peptides, which were verified by ELISA, proteomics analysis revealed a number of coeluted proteins with differential abundance between AD and non-AD eluents. Among AD-enriched proteins, we detected GFAP, CD44 and neurofilament proteins, including neurofilament light, medium and heavy (NEFL, NEFM, and NEFH, respectively), which are markers of axonal degeneration, consistent with previous observations [60]. Additionally, a number of proteins showed decreased abundance in the AD samples, including FKBP4, PTPRZ1 and CBR1, consistent with a previous study showing low levels of these proteins in the soluble fraction of neocortical samples from patients with advanced AD [61]. Some of these coeluted proteins might in turn contribute to a specific microenvironment responsible for the generation of stable misfolded Aβ species that are resistant to proteinase K [58] and/or serve as important cofactor(s) enhancing Aβ neuritotoxicity. Consistent with this suggestion, we detected serum amyloid P-component (SAP; gene name *APCS*), C1q, APOD, agrin (AGRN), and HSPB1 among AD-enriched proteins, which were previously shown to be associated with Aβ plaques [62–66]. Specifically, because C1q directly binds to Aβ, it can trigger activation of the complement system [67] and thus might be partially responsible for the hippocampal proinflammatory response observed in our study. In addition, AD fractions were enriched with proteins such as kallikrein-4 (KLK4), flotilin-2 (FLOT2), RTN3 and RTN4 (the latter is also known as Nogo; Fig. 1C), which were previously shown to alter neuronal APP processing [68–71]. Furthermore, some proteins enriched in AD samples were shown to trigger Aβ fibrillation, such as syndecan-4 (SDC4) [72] and agrin [65], or, in contrast, increase the solubility of amyloid peptides such as SAP [73] and inhibit Aβ aggregation, such as HSPB1 [74] and MT3 [75]. Thus, despite the suggested main role of soluble AβOs in the onset of AD-related pathology, additional proteins might contribute to a high propensity of the neurotoxic effects of AβOs.

In contrast to multiple studies using synthetic amyloid peptides (including our own [21]), cerebral infusion of natural soluble AβOs in WT rats or mice was previously used mainly to establish an acute model of AD and investigate the immediate effects of AβOs on cognitive functions [7, 14]. Specifically, behavioral tests on rats treated with native human AβOs have been limited to one or two cognitive tasks, including novel object recognition, open field, passive avoidance or alternating level cycle ratio (ALCR) tests, which were performed within 24 h to 7 days after the injections and showed a rapid onset and transient amnestic effects of soluble AβOs [7, 14]. Among only a few prolonged studies, Cleary et al. [7] showed that repetitive weekly infusions of AβOs for 4 weeks did not worsen the overall performance of rats on the ALCR test [7]. Nevertheless, the longitudinal effects of these infusions on cognitive function and neurodegenerative alterations have received little attention. In the present study, a battery of behavioral tests, including the EPM, SRT, MWM, and Y-maze, were performed on rats within 6 weeks after the injection of human-derived soluble AβOs to assess the persistent effects of native AβO infusion on behavior, learning and memory. The anxiety of AD rats was not affected in general. However, they showed a transient increase in exploratory behavior by spending more time in the center of the EPM, similar to 5xFAD mice, who developed an age-dependent decreased anxiety phenotype [76]. Compared to controls, AD rats showed no cognitive impairment in spatially demanding tasks, i.e., MWM and Y-maze, consistent with data from Tg AD mice and rats, where no deficits in spatial memory, as assessed using MWM, were observed in the early stages of AD pathology [77]. The hippocampus is critically involved in the formation of spatial memory. The observed lack of changes in the number of pyramidal neurons and moderate region-dependent synaptic destruction in this study might account for the unaltered spatial performance of AD rats. Our results suggest that the observed moderate changes in the hippocampal structure, including microgliosis and increase in apoptotically dying cells, were unable to promote significant hippocampus-dependent spatial memory deficits in AD rats. In addition to spatial memory, the hippocampus and EC are known to be involved in nonspatial memory processing [78, 79]. In humans and rats, the EC is anatomically and functionally connected to all regions of the hippocampus [80]*.* Projections of neurons from the LEC to the DG are necessary for social memory retrieval, particularly for short-term recognition memory [81], and these axons are known to be significantly affected in AD pathology [52]. As the EC being one of the earliest affected cortical areas during AD progression [42], the EC-to-hippocampal synaptic connection has previously been shown to be sensitive to soluble Aβ species and pTau [43, 44]. The significant decrease in the LEC volume accompanied by microgliosis and higher number of cleaved Caspase-3^+^-cells and the hippocampal neuroinflammatory response observed in our study might explain the decline in social recognition memory in the AD rats. The observed correlations between social memory (RR index) and the LEC volume or the level of hippocampal IBA1-IR provide further strong support for this hypothesis. Further investigation of neurodegenerative changes occurring within the EC during early stages of AD is required.

Multiple lines of evidence suggest that deficits in synaptic plasticity reflected by a loss or dysfunction of synapses constitute one of the early pathological changes during AD progression [82, 83] and are postulated to be induced by soluble Aβ [14], which binds directly to pre- and postsynaptic areas [24, 84]. Our quantitative IHC analysis of two presynaptic markers, SYP and VGLUT1, revealed a decrease in SYP-IR within the CA1 region of the AD rat hippocampus. This finding is consistent with observations in humans, where a decrease in hippocampal SYP levels was detected in early stages of AD progression and correlated with the degree of cognitive impairment [82, 85, 86]. In contrast, we observed no differences in VGLUT1 IHC labeling and gene expression, as assessed using qPCR, between groups. Notably, the altered level of glutamatergic activity was previously associated with the disease stage; glutamatergic activity was high in individuals with early/mild stages but decreased during disease progression [87]. Specifically, the increase in glutamatergic terminals together with hippocampal hyperactivity were previously shown in humans with mild AD [88], and increased excitability of pyramidal neurons at early stages of AD was reported in mice [77, 89], where hippocampal VGLUT1 expression was upregulated [53]. Thus, the eventual effect of the AβO infusion on hippocampal VGLUT1 levels might be masked by a potential increase in excitability occurring along with early AD-related pathological changes. Further analysis, e.g., examining glutamate concentrations in live rats following the infusion, is needed to confirm this hypothesis. In addition, the observed increase in the expression of the postsynaptic density markers *PSD95* and *5HTR2A* further indicates a dysregulated synaptic system in AD rats.

Neuroinflammation is one of the early hallmarks of AD and is dynamically implicated in the progression of the disease [48, 49]. Brain region- and time-dependent microglial activity [90] and altered cytokine levels in CSF [91, 92] and brain parenchyma [93, 94] have been described in patients with mild AD and in animal models of AD [46]. Here, we showed signs of neuroinflammation manifested at the histological and molecular levels in AD rat brains at 6 weeks after the infusion. This time point was chosen to cover the period of switching from acute to chronic pathology and to fulfill the gap of knowledge regarding the tissue pathology between short-term (24 h–7 days) responses to AβO infusion [7, 14, 25] and well-described time-dependent AD progression for various Tg animals, commonly started from 3 months of age [46]. The region-dependent activation of hippocampal microglia, as reflected by increased levels of IBA1-IR within CA, was also confirmed by the upregulation of microglia-specific genes, *P2RY12* and *TMEM119,* and inflammation- and oxidative stress-associated genes, such as *IL6* and *HIF1a*, respectively. Similarly, previous studies showed upregulated expression of the motility-related microglial protein P2RY12 in response to Aβ; however, in advanced AD stages, its mRNA level was downregulated [50, 95]. Specifically, P2RY12 is responsible for directed motility, which occurs in early stages of microglial activation, whereas reactive proinflammatory microglial cells exhibit decreased levels of *P2RY12* expression [96]. Likewise, the mRNA level of another resident microglial marker, *TMEM119* [50], was reported to be upregulated in human AD brains [97], consistent with our results. Meanwhile, *TMEM119* was downregulated in Tg mice with advanced AD [47]. Thus, the altered expression of these two microglial markers might be region- and species-dependent and most likely sensitive to a specific stage of the disease [46]. A key function of microglia, namely, clearance of Aβ aggregates from brain parenchyma, is known to be modulated by inflammatory cytokines, particularly by IL6, which facilitates the clearance of soluble Aβ and attenuates the deposition of amyloid plaques in the early disease stage [98]. Therefore, the observed upregulation of *IL6* in this study is consistent with previous findings. In addition, the observed upregulation of *EIF2AK2*, which encodes a pro-apoptotic stress-activated protein kinase R (PKR), and higher number of cleaved caspase-3^+^-cells further indicates the ongoing stress and inflammatory responses in the hippocampus of AD rats. PKR is known to phosphorylate the α subunit of eukaryotic initiation factor 2 (EIF2α), which leads to the inhibition of protein synthesis and eventual cell death [99]. Elevated levels of active PKR and its main target, EIF2α, have been reported in plasma [100], CSF [101] and brains of patients with AD [102]. Our results are supported by a previous observation that an i.c.v. injection of Aβ peptides in monkeys induces PKR and EIF2α phosphorylation along with synaptic loss and memory impairments through a mechanism associated with inflammation [103]. Thus, we suggest that our model mimics the microglial response observed in early stages of hippocampal AD pathology and provides strong support for the inflammatory processes that occur before plaque formation. Further in-depth mechanistic investigations are required to determine whether neuroinflammation is a cause or a consequence of Aβ pathology.

Aiming to further investigate AD-related hallmarks at the transcriptional level, we detected the significant upregulation of *APOE*, *B2M*, *EIF2AK2* and a tendency for *GSK3β,* each of which is known as a risk factor and/or hallmark of AD [104–106]. Previously, APOE, the major lipid transporter in the brain, and B2M, a marker of phagocytosis, were not functionally linked with microglia; however, a recent single-cell RNA-seq analysis showed that these two genes were upregulated in microglia, particularly during the early stage of AD pathology in 5xFAD mice [47]. Furthermore, APOE was shown to directly modulate the response of microglia to Aβ, since the deletion of this gene substantially reduced the number of Aβ-activated microglia [46]. The gradual shift of the microglial transcriptional profile from a homeostatic to a disease-associated phenotype during disease progression was recently reported at single-cell resolution [46, 47]. Interestingly, our qPCR results showed no upregulation of microglial genes related to more advanced stages of AD, such as *TREM2*, *CST7* and *LPL*.

Together, we showed that hippocampal microglia were polarized toward an activated state in the current model 6 weeks after the intracerebral infusion of AD tissue extract, which is characterized by increased phagocytic and motile activity and sustained inflammatory response. Acknowledging that this study has a limitation due to the single time point used for tissue collection and thus does not allow us to explore the temporal relationship between different markers investigated here, we nevertheless suggest that the chosen time of sampling (6 weeks after the infusion) is still matched to the early plaque-free stage of AD pathology.

Different protocols for the isolation of native soluble Aβ species from mechanically disrupted brain tissue were implemented in previous studies using various aqueous extraction buffers, e.g., TBS, PBS, Hank’s balanced salt solution (HBSS), artificial CSF and Ham’s F12 medium [11, 23, 61]. A recent comparative analysis of differently prepared human AD brain tissue extracts showed that neurotoxic amyloid species are efficiently eluted by soaking tissue in an extraction buffer [23]. Therefore, we included the gentle elution step in the preparation of human tissue protein extracts in the present study, incubating the disrupted tissue on ice for 30 min in PBS followed by two sequential centrifugation steps. Hence, the residual supernatant had a higher load of Aβ species in samples eluted from human AD brain tissues compared to non-AD cortices. However, the analysis of postinoculated rat brain tissues using ELISA and IHC with 4G8 and 6E10 anti-β amyloid antibodies, respectively, both of which are specific for N-terminal epitopes of human Aβ, showed that neither diffuse nor dense-core plaques were present in the brain 6 weeks after the infusion. More than one factor is likely responsible for eliminating exogenous Aβ species from the brain parenchyma, including the time elapsed after the inoculation, initial amount of injected material and active endogenous repair processes. Notably, in contrast to humans and a few other species, such as dogs and nonhuman primates [16], WT mice and rats are devoid of spontaneous and progressive accumulation of Aβ plaques, at least partially due to three different amino acid residues in the APP sequence [107, 108]. Hence, consistent with this finding, our data illustrate that exogenously applied native soluble Aβ species did not induce plaque formation in the WT rat brain, confirming previous results obtained with WT mice [11, 12, 109]. Nevertheless, we observed a decline in social memory associated with early stages of inflammatory processes and reorganization of synaptic networks in the hippocampus following an i.c.v infusion of AβΟ extracts. We speculate that these changes might be induced by resilient AβO complexes that were present in the brain, yet at concentrations below histological and biochemical detection levels. This suggestion is consistent with the known remarkable potency of natural AβOs [7] and with recent data showing that exogenous human-derived Aβ species are present in the recipient brain at levels below detection and still retain their pathogenic properties [110]. However, we do not exclude the possibility that other coeluted proteins might be implicated in the observed behavioral and histological effects. Nonetheless, we propose that the inoculation of WT rats with native AβOs might be an animal model well suited for modeling the initial plaque-free stages of AD-related pathology, particularly for the elucidation of the early steps of this disease at cognitive and structural levels.

In conclusion, a cerebral infusion of native AβOs decreased social memory and decreased the LEC volume in rats 6 weeks after inoculation in the absence of amyloid seeds and without changes in spatial memory. The infusion of AD human brain tissue extracts resulted in a neuroinflammatory response, increased cell death and synaptic reorganization and was associated with a decline in short-term social memory. Further studies are necessary to evaluate the early events of neuronal lesions induced by infused native oligomeric Aβ.

## Supplementary Information

**Additional file 1. Fig. S1**: (A) Data from the EPM test. Number of entries in open (left panel) and closed arms (center panel) and in the central square (right panel) analyzed for each 2.5 min time interval. Data are presented as the means ± SEM. *: p < 0.05. In the MWM test, control and AD rats showed a similar (B) latency to the platform and similar (C) total distance traveled during acquisition training (left panel) and probe tests (right panel). (A–C) n = 12 rats per group.

**Additional file 2. Fig. S2**: Comparison of the voxel intensity in (A) the whole brain, (B) hippocampus, and (C) EC between control and AD rats. (D) Stereological quantification of neurons in the CA region of the hippocampus. The results from Giemsa staining are visualized as the number of neurons per mm3. Neurons were stereologically counted in the CA1 and CA3 areas of the hippocampus. Data are presented as the means ± SEM, n = 3 rats per group. (E) Pearson’s correlation analysis indicates a negative correlation between the RR index and the volume of LEC and (F) the RR index and the total brain volume.

**Additional file 3. Fig. S3**: (A) Representative images of row MRI T2W image with entorhinal cortex (yellow arrows) and further caudal from that a cochlea (red arrows). (B) Representative images of labeling with the anti-Aβ 6E10 antibody showing no plaques in the brains of control rats injected with human non-AD brain tissue extracts (top panels) and WT F344 rats (bottom panels). Scale bar, 300 µm.

**Additional file 4. Fig. S4**: Relative mRNA expression of AD markers in the hippocampus of AD and control rats (n = 6 per group) measured using qPCR. Data were normalized to two reference genes: ACTB and RPL13A. TREM2: triggering receptor expressed on myeloid cells 2, CST7: cystatin F, LPL: lipoprotein lipase, IL1b: interleukin-1β, TNFa: tumor necrosis factor-α.

**Additional file 5. Table S1**: Demographic data for human brain samples from the prefrontal cortex.

**Additional file 6. Table S2**: List of primers used for qPCR.

**Additional file 7. Table S3**: Complete lists of the identified proteins for AD and control brain extracts using quantitative mass spectrometry-based proteomics.

## Data Availability

The proteomics data are available at the PRIDE repository with the dataset identifier PXD035962.

## Abbreviations

5HTR2A: Serotonin 2A receptor
Aβ: Amyloid beta
ACTB: Actin beta
AD: Alzheimer’s disease
AGRN: Agrin
APOE: Apolipoprotein E
APP: Amyloid precursor protein
B2M: β-2-Microglobulin
CA: Cornu ammonis
CST7: Cystatin F
DG: Dentate gyrus
EIF2AK2: Eukaryotic translation initiation factor 2 alpha kinase 2
EPM: Elevated plus maze
FAD: Familial Alzheimer’s disease
GFAP: Glial fibrillary acidic protein
GSK3β: Glycogen synthase kinase 3 beta
HIF1a: Hypoxia inducible factor 1 subunit alpha
IBA1: Ionized calcium binding adapter molecule 1
IHC: Immunohistochemistry
IL6: Interleukin 6
KLK4: Kallikrein-4
LEC: Lateral entorhinal cortex
LPL: Lipoprotein lipase
LTP: Long-term potentiation
MEC: Medial entorhinal cortex
MEMRI: Manganese-enhanced MRI
MWM: Morris water maze
MRI: Magnetic resonance imaging
NMDAR: *N*-methyl-d-aspartate receptor
P2RY12: Purinergic receptor P2Y12
PSD95: Postsynaptic density protein 95
qPCR: Quantitative real-time PCR
ROI: Region of interest
RPL13a: Ribosomal protein L13 alpha
SEM: Standard error of the mean
SDC4: Syndecan-4
SRT: Social recognition test
SYP: Synaptophysin
SYT1: Synaptotagmin-1
TMEM119: Transmembrane protein 119
TBS: Tris-buffered saline
TREM2: Triggering receptor expressed on myeloid cells 2
VGLUT1: Vesicular glutamate transporter 1
