# Supplementary figures and images for: Modeling the early stages of Alzheimer’s disease by administering intracerebroventricular injections of human native Aβ oligomers to rats
Source: Acta Neuropathol Commun. 2022 Aug 16;10:113. doi: 10.1186/s40478-022-01417-5 (PMC9380371; doi:10.1186/s40478-022-01417-5)

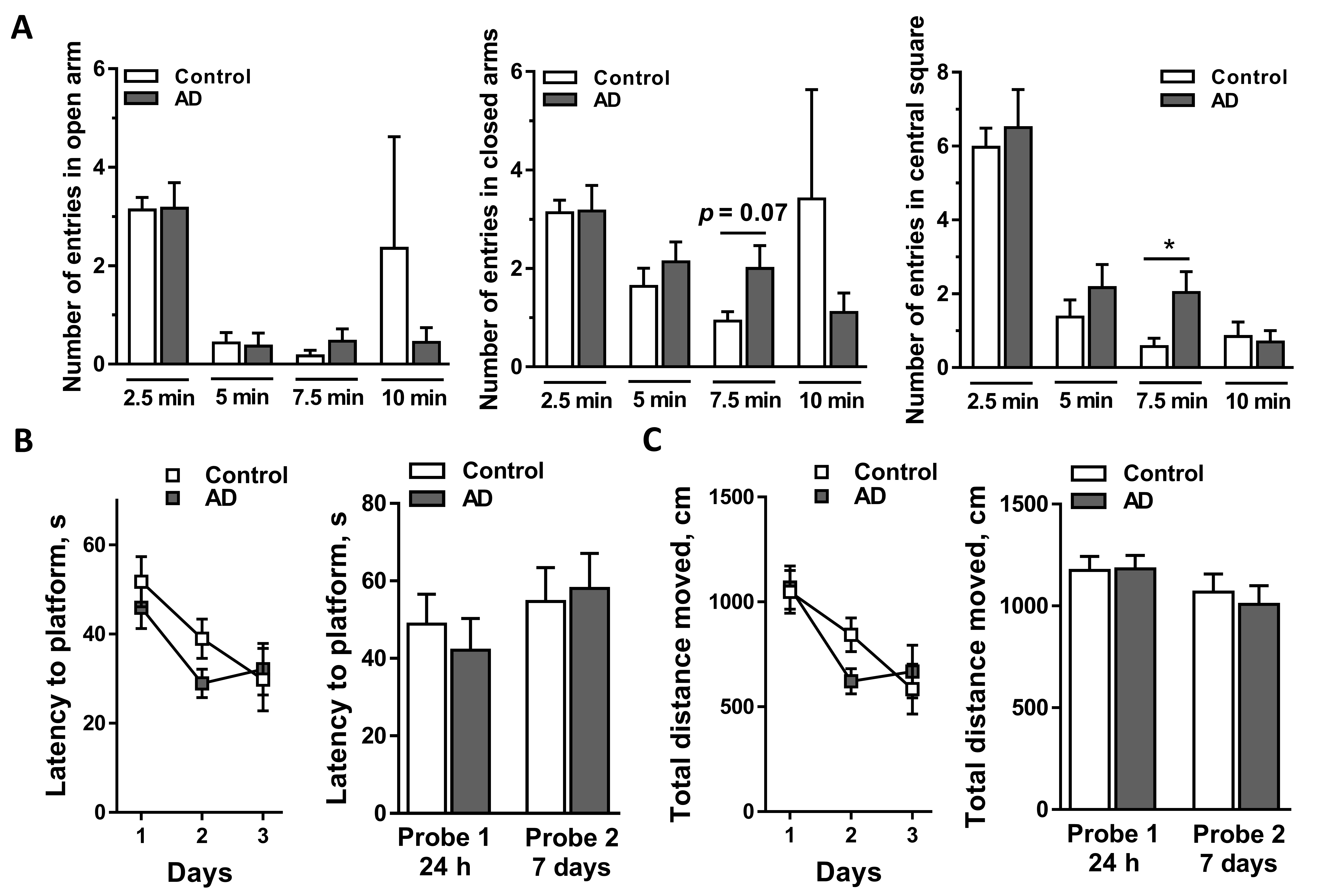

Supplement: Supplementary file 1 — Additional file 1. Fig. S1: (A) Data from the EPM test. Number of entries in open (left panel) and closed arms (center panel) and in the central square (right panel) analyzed for each 2.5 min time interval. Data are presented as the means ± SEM. *: p < 0.05. In the MWM test, control and AD rats showed a similar (B) latency to the platform and similar (C) total distance traveled during acquisition training (left panel) and probe tests (right panel). (A–C) n = 12 rats per group. [file 40478_2022_1417_MOESM1_ESM.tif]

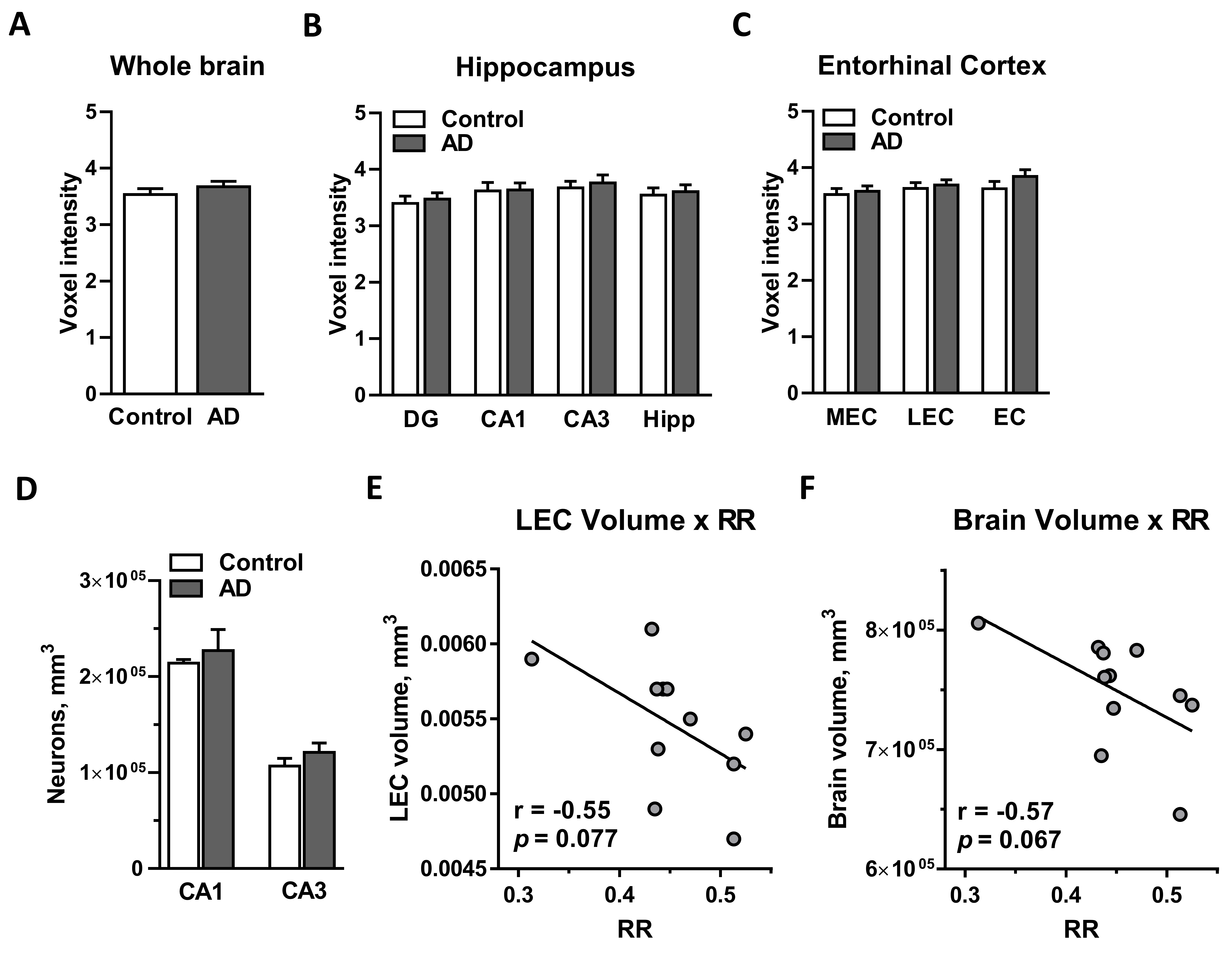

Supplement: Supplementary file 2 — Additional file 2. Fig. S2: Comparison of the voxel intensity in (A) the whole brain, (B) hippocampus, and (C) EC between control and AD rats. (D) Stereological quantification of neurons in the CA region of the hippocampus. The results from Giemsa staining are visualized as the number of neurons per mm3. Neurons were stereologically counted in the CA1 and CA3 areas of the hippocampus. Data are presented as the means ± SEM, n = 3 rats per group. (E) Pearson’s correlation analysis indicates a negative correlation between the RR index and the volume of LEC and (F) the RR index and the total brain volume. [file 40478_2022_1417_MOESM2_ESM.tif]

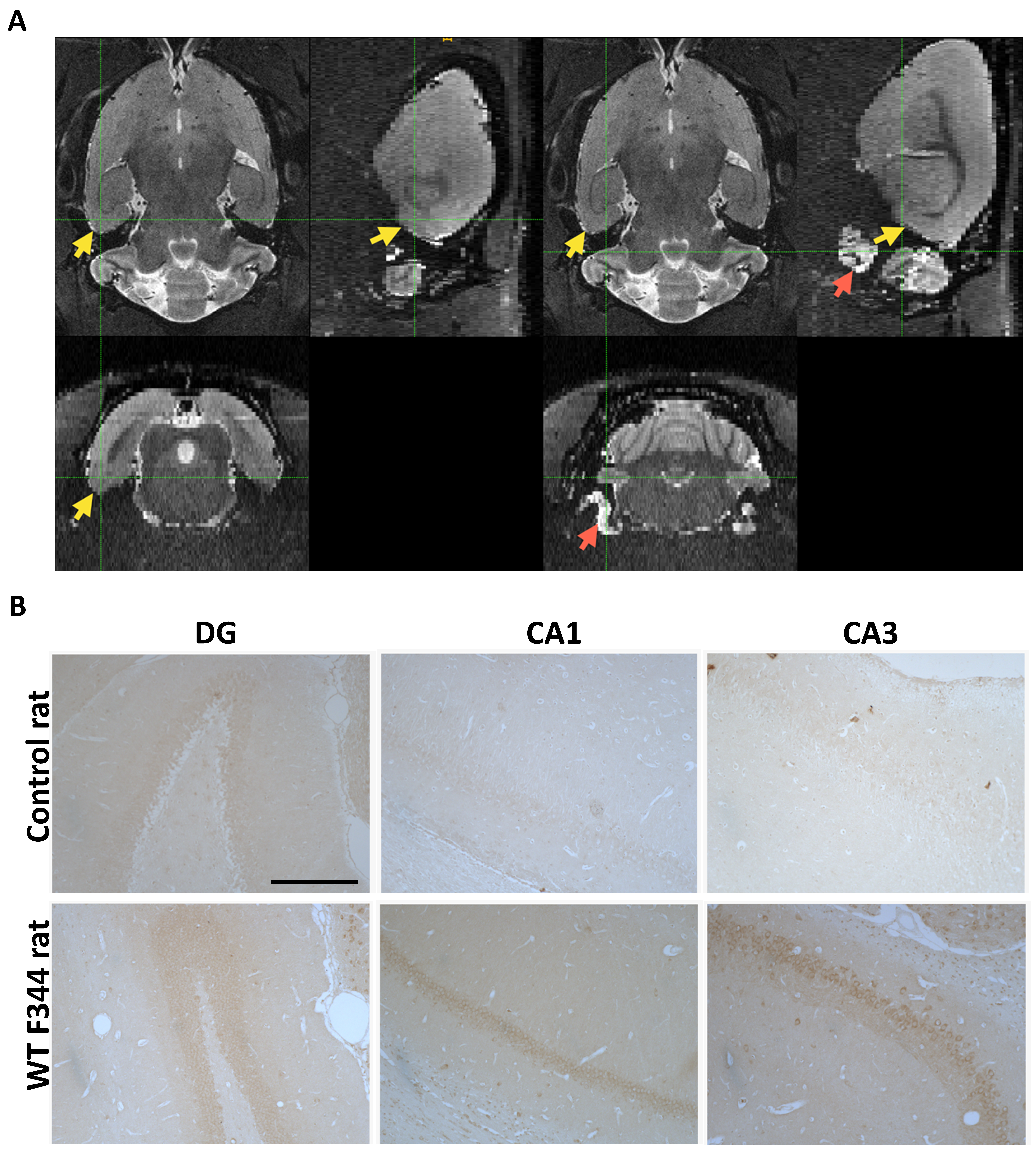

Supplement: Supplementary file 3 — Additional file 3. Fig. S3: (A) Representative images of row MRI T2W image with entorhinal cortex (yellow arrows) and further caudal from that a cochlea (red arrows). (B) Representative images of labeling with the anti-Aβ 6E10 antibody showing no plaques in the brains of control rats injected with human non-AD brain tissue extracts (top panels) and WT F344 rats (bottom panels). Scale bar, 300 µm. [file 40478_2022_1417_MOESM3_ESM.tif]

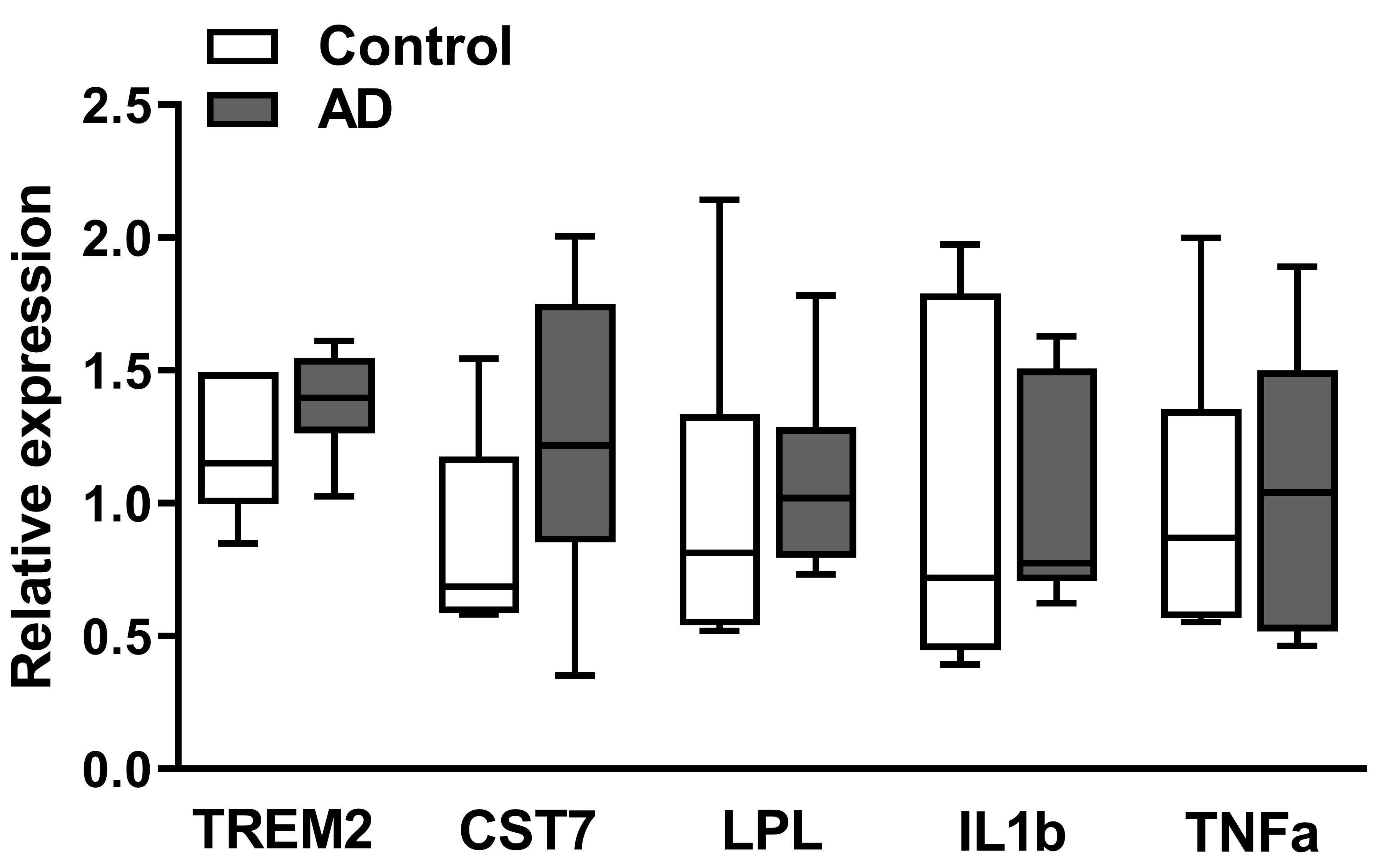

Supplement: Supplementary file 4 — Additional file 4. Fig. S4: Relative mRNA expression of AD markers in the hippocampus of AD and control rats (n = 6 per group) measured using qPCR. Data were normalized to two reference genes: ACTB and RPL13A. TREM2: triggering receptor expressed on myeloid cells 2, CST7: cystatin F, LPL: lipoprotein lipase, IL1b: interleukin-1β, TNFa: tumor necrosis factor-α. [file 40478_2022_1417_MOESM4_ESM.tif]
