# Supplementary material for: Modeling the early stages of Alzheimer’s disease by administering intracerebroventricular injections of human native Aβ oligomers to rats
Source: Acta Neuropathol Commun. 2022 Aug 16;10:113. doi: 10.1186/s40478-022-01417-5 (PMC9380371; doi:10.1186/s40478-022-01417-5)
Supplement: Supplementary file 5 — Additional file 5. Table S1: Demographic data for human brain samples from the prefrontal cortex. [file 40478_2022_1417_MOESM5_ESM.docx]

**Supplementary Table S1:** Demographic data of human brain samples from prefrontal cortex

| Samples | Age (years) | Sex | Braak staging | PMI (hh:mm) |
| --- | --- | --- | --- | --- |
| NC1 | 79 | M | 1 | 7:40 |
| NC2 | 78 | F | 1 | 4:50 |
| NC3 | 75 | M | 1 | 6:20 |
| NC4 | 86 | M | 2 | 5:30 |
| NC5 | 91 | F | 3 | 5:20 |
| AD1 | 84 | F | 5 | 3:50 |
| AD2 | 72 | F | 6 | 2:51 |
| AD3 | 87 | M | 4 | 3:45 |
| AD4 | 66 | F | 6 | 6:30 |
| AD5 | 69 | F | 5 | 3:50 |
| AD6 | 88 | F | 4 | 12:15 |
| *p-value* | 0.442^#^ | 0.137^¤^ | *0.002^§^* | 0.781^§^ |
| NC: Non-demented control; AD: Alzheimer’s disease patients; PMI: Post-mortem interval. Samples autopsied from gyrus medialis frontalis. ^#^: Student’s *t*-test. ^¤^: Chi-squared test. ^§^: Mann-Whitney U test. *P* < 0.05 was considered significant. | | | | |
