# Supplementary material for: Modeling the early stages of Alzheimer’s disease by administering intracerebroventricular injections of human native Aβ oligomers to rats
Source: Acta Neuropathol Commun. 2022 Aug 16;10:113. doi: 10.1186/s40478-022-01417-5 (PMC9380371; doi:10.1186/s40478-022-01417-5)
Supplement: Supplementary file 6 — Additional file 6. Table S2: List of primers used for qPCR. [file 40478_2022_1417_MOESM6_ESM.docx]

**Supplementary Table S2:** List of primers used for qPCR

| **Gene symbol** | **Gene name** | **Primer sequences (5’ to 3’)** | **Product size (bp)** |  |
| --- | --- | --- | --- | --- |
| **Reference genes** | | | |  |
| ACTB | Actin Beta | **F:** TCTGTGTGGATTGGTGGCT  **R:** TCATCGTACTCCTGCTTGCT | 80 |  |
| RPL13A | Ribosomal Protein L13a | **F:** AGCAGCTCTTGAGGCTAAGG  **R:** GGGTTCACACCAAGAGTCCA | 102 |  |
| **Inflammation- and AD-related genes** | | | |  |
| P2RY12 | Purinergic receptor P2Y12 | **F:** TTGCACGGATTCCCTACACC  **R:** GGGTGCTCTCCTTCACGTAG | 90 |  |
| TMEM119 | Transmembrane Protein 119 | **F:** GCTACGCTTTCTTCACGTTGC  **R:** AACCAATCAGGAAGTGGGGT | 130 |  |
| IL1β | Interleukin 1 beta | **F:** CACCTCTCAAGCAGAGCACAG  **R:** GGGTTCCATGGTGAAGTCAAC | 79 |  |
| IL6 | Interleukin 6 | **F:** AGCGATGATGCACTGTCAGA  **R:** GGAACTCCAGAAGACCAGAGC | 127 |  |
| HIF1α | Hypoxia-inducible Factor 1 α | **F:** CAACTGCCACCACTGATGAA  **R:** TGGGTAGAAGGTGGAGATGC | 88 |  |
| B2M | Beta-2-Microglobulin | **F:** CCCACCCTCATGGCTACTTC  **R:** GATGAAAACCGCACACAGGC | 157 |  |
| APOE | Apolipoprotein E | **F:** AAGCTTGGGCTGGCGATTCA  **R:** CCCACAGAGCCTTCATCTTCC | 72 |  |
| EIF2AK2 | Eukaryotic Translation Initiation  Factor 2 α kinase 2 | **F:** CAGAAGCGACAGTGGTCGG  **R:** GGCCATTTTTCTTCCCCGGT | 129 |  |
| GSK3β | Glycogen Synthase Kinase 3 β | **F:** ACTCTACCTGAACAGCCCCA  **R:** AACGTGACCAGTGTTGCTGA | 85 |  |
| TNFα | Tumor necrosis factor | **F:** AAATGGGCTCCCTCTCATCAGTTC  **R:** TCCGCTTGGTGGTTTGCTACGAC | 111 |  |
| TREM2 | Triggering Receptor Expressed on Myeloid Cells 2 | **F:** TTGGCATACTCTCCCCTCCA  **R:** CACCAGTATTCCTGCTCCCG | 105 |  |
| LPL | Lipoprotein Lipase | **F:** CCAGCTGGGCCTAACTTTGA  **R:** GGAAAGTGCCTCCATTGGGA | 169 |  |
| CST7 | Cystatin F | **F:** GCTGCCACTGACTTCTGTCT  **R:** CACTGGTAAGCAGGCCTTGA | 124 |  |
| **Synaptic genes** | | | |  |
| SYP | Synaptophysin | **F:** AACAAAGGGCCTATGATGGA  **R:** CCAGGTTCAGGAAGCCAAA | 232 |  |
| SYT1 | Synaptotagmin 1 | **F:** GACGATGCTGAAACCGGACT  **R:** CAGCCTGGATGATTCCCACC | 136 |  |
| 5-HT2A | Serotonin 2A Receptor | **F:** CCACAGCCGCTTCAACTC  **R:** GCAGCTCCCCTCCTTAAAGA | 136 |  |
| DLG4 | Discs Large MAGUK  Scaffold Protein 4 (PSD-95) | **F:** GCC CTG TTT GAT TAC GAC AA  **R:** CTCATAGCTCAGAACCGAGT | 270 |  |
| GRIN1 | Glutamate Ionotropic Receptor NMDA Type 1 (NMDAR) | **F:** AGTGGGCATCTACAATGGT  **R:** TCTGGTGGACATCTGGTATC | 103 |  |
| SLC17A7 | Solute Carrier Family 17  Member 7 (VGLUT1) | **F:** TGGGTTTCTGCATCAGCTTTG  **R:** TGTACTGTTGTTGACCATGGATACG | 74 |  |
